# Novel tetronamides derived from mucobromic acid: synthesis, structural elucidation, DFT calculations and phytotoxic activity

**DOI:** 10.1002/ps.71179

**Published:** 2026-08-06

**Authors:** Kamylla CF Faria, Vitor C Baia, Elson S Alvarenga

**Affiliations:** ^1^ Department of Chemistry Universidade Federal de Viçosa Viçosa Brazil

**Keywords:** herbicide, menthol, mucobromic acid, Tetronamides, theoretical calculations

## Abstract

**BACKGROUND:**

Motivated by the growing demand for increased food production and the need to identify new, more selective and effective herbicides in view of the resistance developed by plants to traditional herbicides, we synthesized six novel tetronamides derived from mucobromic acid and menthol. The structures of these compounds were characterized by spectroscopic techniques, complemented by theoretical calculations based on Density Functional Theory (DFT). In addition, we evaluated the phytotoxic activity of these substances in species such as *Cucumis sativus*, *Solanum lycopersicum*, *Sorghum bicolor* L. Moench, *Urochloa brizantha* and *Bidens pilosa*.

**RESULTS:**

The acyclic form of mucobromic acid was activated by (1*S*)‐(+)‐10‐camphorsulfonic acid (CSA) for the enantioselective synthesis of compound 1b, which served as a starting point for the aza‐Michael addition/elimination reaction on the butenolide ring. In this reaction, the carbon at the C4 position was activated, allowing the nucleophilic attack by different substituted anilines, giving rise to the new tetronamides 2b‐7b. The relative configuration of the chiral centers of these substances was defined as *SRSR* based on spectroscopic analysis, theoretical calculations and the probabilistic methods DP4, DP4+, Linear Regression (R^2^), and Corrected Mean Absolute Error (CMAE). The bioassays performed indicated significant inhibition of the growth of roots and shoots of *U*. *brizantha*.

**CONCLUSION:**

These results confirm the potential of these tetronamides as selective phytotoxic agents and pave the way for the future development of more effective and sustainable herbicides, contributing to intelligent weed management and increased agricultural productivity. © 2026 The Author(s). *Pest Management Science* published by John Wiley & Sons Ltd on behalf of Society of Chemical Industry.

## Graphical abstract

The acyclic mucobromic acid, activated by (1S)‐(+)‐10‐camphorsulfonic acid, afforded the chiral compound **1b**, precursor for aza‐Michael addition/elimination on the butenolide yielding tetronamides **2b‐7b**. Their relative SRSR configuration was confirmed by the spectrometric methods, DFT calculation and statistical methods. Bioassays showed significant inhibition of *U. brizantha* root and shoot growth.

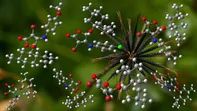

## 1 INTRODUCTION

Weed control is a fundamental practice in modern agriculture, as these species adversely affect crop growth and development by competing for essential resources such as light, water, nutrients, and physical space, ultimately compromising agricultural productivity.^1^, ^2^, ^3^ However, the extensive and often indiscriminate use of herbicides over the decades has raised significant environmental and toxicological concerns‐particularly regarding soil and water contamination, the evolution of resistance in invasive species, and adverse effects on non‐target organisms, including beneficial insects, soil microorganisms, and humans.^4^, ^5^, ^6^ In this context, the development of more selective and effective agrochemicals that specifically target molecular pathways in weeds‐while minimizing harm to other components of the agricultural ecosystem‐has become increasingly urgent. The search for novel molecules with innovative mechanisms of action, combined with the application of rational synthesis approaches and targeted bioassays, represents a promising strategy for promoting more sustainable and safer agricultural practices.^7^, ^8^, ^9^

Our research group has been dedicated to identifying viable alternatives to traditional pesticides through rational design and structural planning of new bioactive substances.^10^, ^11^, ^12^, ^13^, ^14^, ^15^, ^16^, ^17^, ^18^, ^19^, ^20^ In this approach, we prioritize synthetic routes with lower environmental impact and utilize advanced computational tools to predict the modes of action of the synthesized compounds, as well as to characterize them with greater precision.^21^, ^22^, ^23^, ^24^, ^25^, ^26^, ^27^, ^28^, ^29^, ^30^, ^31^, ^32^, ^33^, ^34^, ^35^, ^36^

Theoretical calculations based on Density Functional Theory (DFT) have established themselves as a powerful computational approach for the structural elucidation of organic compounds. Among its various applications, the prediction of chemical shifts in ^13^C and ^1^H Nuclear Magnetic Resonance (NMR) spectroscopy is particularly noteworthy. To evaluate the reliability of these theoretical predictions, a range of statistical methods has been employed – ranging from simpler metrics such as Mean Absolute Error (MAE), Corrected Mean Absolute Error (CMAE), the linear correlation coefficient (R^2^), and Root Mean Square Deviation (RMSD), to more sophisticated techniques like CP3, DP4, and its enhanced version, DP4+, which allow for the probabilistic identification of the most likely structure among different candidate molecules.^11^, ^37^, ^38^

The CP3 method was developed to assign two sets of experimental data to two possible candidate structures,^39^ while DP4 was designed to determine, among two or more candidates, the structure that best correlates with a single set of experimental NMR data.^40^, ^41^ In these methods, ^13^C and ^1^H NMR data can be used independently or in combination. However, the combined use of both nuclei typically yields more reliable results, especially in cases where the separate analysis of each dataset fails to clearly distinguish between structural candidates.^42^

To enhance the accuracy of the original DP4 method, Sarotti and co‐workers developed DP4 + ,^43^, ^44^ which introduced critical improvements to two fundamental aspects of the original approach. Specifically, DP4+ employs a higher level of theory for chemical shift calculations and incorporates both scaled and unscaled data, thereby expanding the statistical foundation of the probabilistic analysis. In its formulation, DP4+ uses 8 and 16 statistical parameters for ^1^H and ^13^C shifts, respectively, which are adjusted according to the hybridization state of the carbon atoms. This refined approach enables more robust and accurate modeling, thereby increasing confidence in the correct structural assignment based on NMR data.

An additional practical advantage of DP4+ over CP3 and the original DP4 lies in its broader accessibility. While CP3 and DP4 require execution within Java‐based programming environments, DP4+ is available as an spreadsheet (.xlsx format), which allows for the direct input of experimental and theoretical data. This user‐friendly interface facilitates intuitive and automated probabilistic calculations, making the method more accessible to researchers who may not be familiar with advanced computational tools. In this context, the integrated use of DFT‐based predictions and statistical analyses represents a powerful strategy for structural validation, particularly in cases where experimental evidence alone is insufficient for unambiguous structure determination.

One of the fundamental strategies in the research and development process for new agrochemicals is the selection of polyfunctionalized molecules capable of efficiently reacting with each other and acting as electrophiles and/or nucleophiles, enabling the construction of new structures with biological potential. Among the broad spectrum of compounds with these characteristics, mucobromic acid and the natural product L‐menthol stand out.

In this work, we describe the synthesis of novel tetronamides derived from mucobromic acid, L‐menthol, and various *meta*‐ and *para*‐substituted anilines, following the simplified design illustrated in Scheme 1. The structures of the synthesized compounds were elucidated using spectrometric techniques supported by theoretical DFT calculations and the DP4 and DP4+ statistical methods. Many synthetic tetronamides are engineered through structural modifications aimed at increasing their stability, selectivity, and efficacy for pharmaceutical and agrochemical applications.^45^, ^46^, ^47^, ^48^, ^49^

**Scheme 1:**
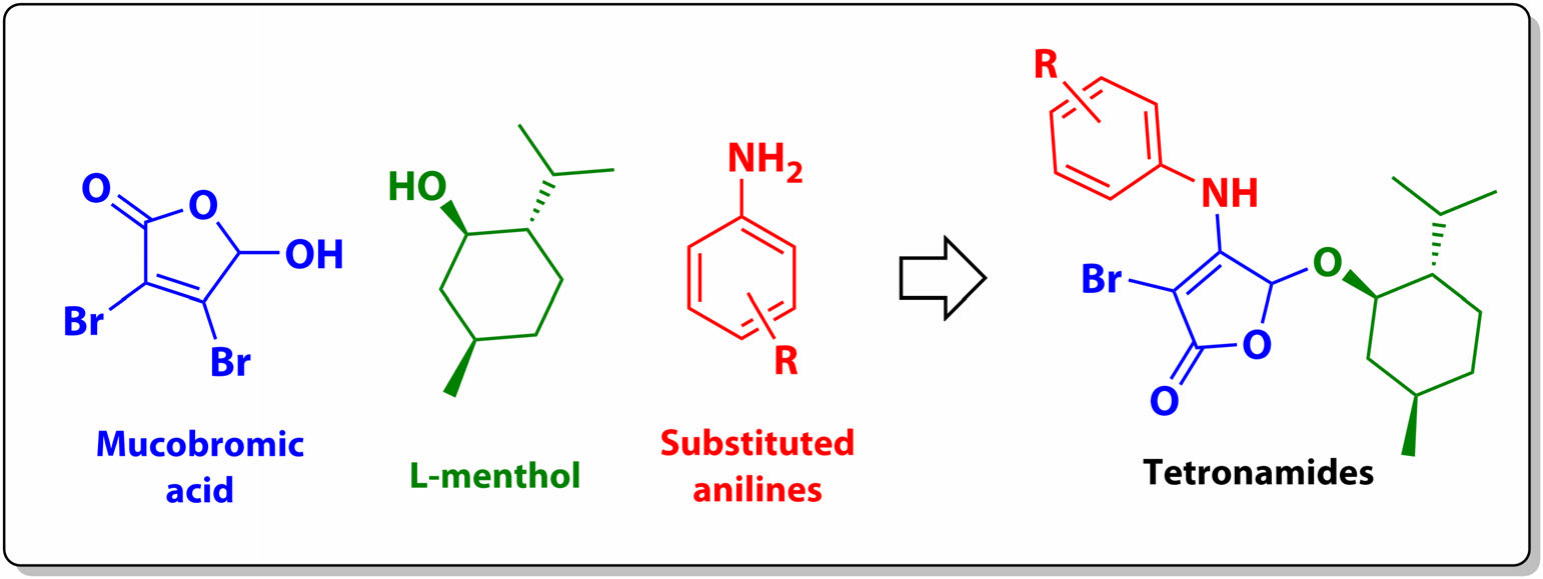
Planning the synthesis of novel tetronamides.

Mucobromic acid was selected as the key synthetic precursor because it is a well‐established electrophilic building block for the preparation of tetronamide derivatives through aza‐Michael addition/elimination reactions. Its highly functionalized butenolide scaffold enables efficient structural diversification while preserving the core heterocyclic framework. This strategy has been successfully employed by several groups. For example, Lattmann and co‐workers^50^ synthesized a series of tetronamides from mucochloric acid and primary amines under acidic conditions, whereas Cunha and co‐workers^51^ prepared structurally diverse tetronamides from reduced mucobromic acid derivatives and secondary amines. The group of Mo^52^ demonstrated that prior acetalization of mucobromic acid with L‐menthol provides a straightforward route to chiral tetronamides. Importantly, Barbosa and co‐workers.^53^ also explored mucobromic acid as a synthetic precursor for rubrolide analogues identified as inhibitors of the photosynthetic electron transport chain, highlighting the relevance of this scaffold for agrochemical discovery. Based on these precedents, mucobromic acid was selected as a versatile platform for generating structurally diverse tetronamides, whereas L‐menthol was employed to introduce a well‐defined chiral framework into the target molecules.

## 2 MATERIALS AND METHODS

### 2.1 General procedures

The melting points were obtained in MQAPF‐302 melting point apparatus and were not corrected. Infrared (IR) spectra were acquired using *Agilent Cary 660 FTIR* spectrophotometer (equipped with GLADI‐ATR, Agilent, Santa Clara, CA, USA) using the attenuated total reflectance (ATR) method. NMR spectra were obtained on *Varian Mercury 300 MHz* and Bruker Avance Core 400 MHz spectrometers using CDCl_3_ as solvent. ^1^H NMR chemical shifts were reported using residual CHCl_3_ signal (*δ* = 7.27 ppm) as reference. ^13^C NMR chemical shifts were recorded using CDCl_3_ (*δ* = 77.0 ppm) as reference. Mass spectra were obtained using Shimadzu (Kyoto, Japan) GC–MS‐QP5050A after 70 eV electron impact ionization. ^1^H and ^13^C NMR, IR and mass spectra of the synthesized target compounds **1–7** can be found in Supplementary Information (SI).

### 2.2 Synthetic procedures

#### 2.2.1 Synthesis of butenolide **1**

In a 150 mL round‐bottomed flask, mucobromic acid (2 mmol, 0.500 g, 1 equiv), (−)‐menthol (2 mmol, 0.312 g, 1 equiv) and (1*S*)(+)‐10‐camphorsulfonic acid (2 mmol, 0.450 g, 1 equiv) were dissolved in toluene (20 mL). A Dean–Stark apparatus fitted with a reflux condenser was attached, and the mixture was heated under reflux (110 °C) for 24 h. Reaction progress was monitored by thin‐layer chromatography (TLC). Upon completion, the toluene was removed under reduced pressure. The residue was dissolved in ethyl acetate with gentle warming, and the acid catalyst was removed by three successive extractions with saturated aqueous NaHCO₃. The organic layer was dried over anhydrous Na_2_SO₄, filtered, and concentrated under vacuum. The crude product was purified by silica‐gel column chromatography (NEON, 230–400 mesh; 25 g; column: 30 cm × 2.06 cm i.d.), eluting with hexane/ethyl acetate (5:1, v/v).

#### 2.2.2 (S)‐3,4‐dibromo‐5‐(((1R,2S,5R)‐2‐isopropyl‐5‐methylcyclohexyl)oxy)furan‐2(5H)‐one **1**

White crystalline solid. TLC: Rf = 0.5 hexane/ethyl acetate (5:1 v/v). mp 141.2–142.1 °C. Yield: 82%. IR (ATR, $\bar{\nu }$/cm^−1^): 2922, 2871, 1749, 1622, 1458, 1310, 1139, 957, 638. ^1^H NMR (300 MHz, CDCl_3_, *δ* = 7.27 ppm): *δ* 5.81 (s, 1H, H5), 3.58 (td, *J =* 10.7, 4.4 Hz, 1H, H1'), 2.32 (qt, *J =* 7.1, 3.6 Hz, 1H, H1”), 2.30–2.17 (m, 1H, H6'x), 1.68 (dp, *J =* 14.2, 3.2 Hz, 2H, H3'x and H4'x), 1.38 (dddt, *J =* 16.3, 13.5, 10.3, 3.2 Hz, 2H, H2' and H5'x), 1.11 (q, *J =* 12.1 Hz, 1H, H6'y), 1.01 (dd, *J =* 12.9, 3.4 Hz, 1H, H3'y), 0.94 (d, *J =* 2.2 Hz, 3H, H4”), 0.92 (d, *J =* 2.6 Hz, 3H, H2”), 0.85 (s, 1H, H4'y), 0.82 (d, *J =* 6.9 Hz, 3H, H3”). ^13^C NMR (100 MHz, CDCl_3_, *δ* = 77.0 ppm): *δ* 164.25 (C2), 143.44 (C4), 118.48 (C3), 104.47 (C5), 84.46 (C1'), 48.03 (C2'), 42.15 (C6'), 33.96 (C4'), 31.62 (C5'), 25.26 (C1”), 22.89 (C3'), 22.07 (C4”), 20.86 (C2”), 15.86 (C3”). m/z (%): 400 ([M + 4] 0,01), 398 ([M + 2] 0,02), 396 ([M^+^.] 0,02), 241 (16), 138 (48), 123 (23), 95 (58), 81 (100), 69 (46), 55 (58), 41 (57).

#### 2.2.3 Synthesis of tetronamides **2**–**7**

In a 150 mL round‐bottom flask, compound **1** (3 mmol, 1 equiv) was dissolved in DMSO (20 mL) under stirring. The corresponding amine (4.5 mmol, 1.5 equiv) was then added, and the reaction mixture was stirred at room temperature for 24 h. Upon completion, 10 mL of 1 M HCl was added, and the mixture was extracted three times with DCM. The combined organic layers was dried over anhydrous sodium sulfate, filtered, and concentrated under reduced pressure using a rotary evaporator. The crude product was purified by column chromatography on silica gel (NEON, 230–400 mesh; 25 g; column dimensions: 30 cm × 2.06 cm), using hexane/ethyl acetate (5:1, v/v) as the eluent.

#### 2.2.4 (S)‐3‐bromo‐4‐((4‐chlorophenyl)amino)‐5‐(((1R,2S,5R)‐2‐isopropyl‐5‐methylcyclohexyl)oxy)furan‐2(5H)‐one **2b**

White crystalline solid. TLC: Rf = 0.5 hexane/ethyl acetate (5:1 v/v). mp 163.2–164.6 °C. Yield: 89%. IR (ATR, $\bar{\nu }$/cm^−1^): 3177, 2957, 2871, 1719, 1634, 1339, 1211, 1135, 926, 501. ^1^H NMR (300 MHz, CDCl_3_, *δ* = 7.27 ppm): *δ* 7.42–7.33 (m, 2H, H3a and H5a), 7.18–7.08 (m, 2H, H2a and H6a), 6.06 (s, 1H, H5), 3.43 (td, *J =* 10.6, 4.5 Hz, 1H, H1'), 2.27–2.17 (m, 1H, H6'x), 1.60 (dddd, *J =* 19.8, 12.3, 5.9, 2.8 Hz, 2H, H3'x and H4'x), 1.36 (dtd, *J =* 13.5, 6.8, 3.1 Hz, 2H, H1” and H5'), 1.17 (dddd, *J =* 12.3, 10.3, 3.4, 2.4 Hz, 1H, H2'), 1.05 (td, *J =* 12.4, 10.9 Hz, 1H, H6'y), 0.91 (d, *J =* 6.5 Hz, 3H, H4”), 0.90–0.77 (m, 2H, H3'y and H4'y), 0.75 (d, *J =* 7.0 Hz, 3H, H2”), 0.50 (d, *J =* 6.9 Hz, 3H, H3”). ^13^C NMR (100 MHz, CDCl_3_, *δ* = 77.0 ppm): *δ* 167.13, (C2) 157.11 (C4), 135.66 (C1a), 132.10 (C4a), 129.69 (C3a and C5a), 124.93 (C2a and C6a), 98.80 (C5), 82.15 (C1'), 81.54 (C3), 48.04 (C2'), 42.59 (C6'), 33.89 (C4'), 31.63 (C5'), 24.98 (C1”), 22.80 (C3'), 22.12 (C4”), 21.12 (C2”), 15.83 (C3”). m/z (%): 443 ([M^+^.] 0,05), 225 (30), 207 (100), 178 (74), 83 (45), 69 (41), 55 (82), 43 (53), 41 (72).

#### 2.2.5 (S)‐3‐bromo‐5‐(((1R,2S,5R)‐2‐isopropyl‐5‐methylcyclohexyl)oxy)‐4‐(p‐tolylamino)furan‐2(5H)‐one **3b**

White crystalline solid. TLC: Rf = 0.5 hexane/ethyl acetate (5:1 v/v). mp 153.9–154.5 °C. Yield: 69%. IR (ATR, $\bar{\nu }$/cm^−1^): 3166, 2951, 2869, 1729, 1636, 1203, 1133, 1039, 949, 505. ^1^H NMR (400 MHz, CDCl_3_, *δ* = 7.27 ppm): *δ* 7.19 (d, *J =* 8.1 Hz, 2H, H3a and H5a), 7.07 (d, *J =* 8.1 Hz, 2H, H2a and H6a), 6.07 (s, 1H, H5), 3.40 (td, *J =* 10.6, 4.5 Hz, 1H, H1'), 2.37 (s, 1H, H7a), 2.26–2.18 (m, 1H, H6'x), 1.64–1.51 (m, 2H, H3'x and H4'x), 1.33 (dqd, *J =* 13.9, 7.7, 6.9, 2.8 Hz, 2H, H1” and H5”), 1.18–1.10 (m, 1H, H2'), 1.09–0.97 (m, 1H, H6'y), 0.90 (d, *J =* 6.5 Hz, 3H, H4”), 0.78 (ddd, *J =* 24.2, 12.2, 2.5 Hz, 2H, H3'y and H4'y), 0.69 (d, *J =* 7.0 Hz, 3H, H2”), 0.43 (d, *J =* 6.9 Hz, 3H, H3”). ^13^C NMR (75 MHz, CDCl_3_, *δ* = 77.0 ppm): *δ* 167.43 (C2), 157.89 (C4), 136.54 (C1a), 134.48 (C4a), 130.14 (C3a and C5a), 123.79 (C2a and C6a), 98.77 (C5), 81.92 (C1'), 80.11 (C3), 48.02 (C2'), 42.62 (C6'), 33.93 (C4'), 31.63 (C5'), 24.75 (C1”), 22.79 (C3'), 22.13 (C4”), 21.19 (C2”), 21.02 (C7a), 15.82 (C3”). m/z (%): 424 ([M + 2] 2,75), 422 ([M^+^.] 2,75), 285 (30), 267 (32), 237 (33), 186 (54), 158 (26), 130 (23), 95 (29), 83 (53), 69 (48), 55 (100), 43 (63), 41 (82).

#### 2.2.6 (S)‐3‐bromo‐5‐(((1R,2S,5R)‐2‐isopropyl‐5‐methylcyclohexyl)oxy)‐4‐(phenylamino)furan‐2(5H)‐one **4b**

White crystalline solid. TLC: Rf = 0.5 hexane/ethyl acetate (5:1 v/v). mp 145.0–145.9 °C. Yield: 71%. IR (ATR, $\bar{\nu }$/cm^−1^): 3181, 2922, 2862, 1727, 1625, 1209, 1125, 1041, 934, 492. ^1^H NMR (400 MHz, CDCl_3_, *δ* = 7.27 ppm): *δ* 7.45–7.36 (m, 2H, H3a and H5a), 7.32–7.23 (m, 1H, H4a), 7.21–7.13 (m, 2H, H2a and H6a), 6.12 (s, 1H, H5), 3.41 (td, *J =* 10.6, 4.5 Hz, 1H, H1'), 2.23 (dddd, *J =* 12.6, 4.9, 3.3, 1.9 Hz, 1H, H6'x), 1.65–1.52 (m, 2H, H3'x and H4'x), 1.37 (ddtd, *J =* 19.2, 13.0, 6.8, 2.9 Hz, 2H, H1” and H5'), 1.22–1.12 (m, 1H, H2'), 1.06 (td, *J =* 12.4, 10.9 Hz, 1H, H6'y), 0.91 (d, *J =* 6.5 Hz, 3H, H4”), 0.88–0.75 (m, 2H, H3'y and H4'y), 0.71 (d, *J =* 7.0 Hz, 3H, H2”), 0.43 (d, *J =* 6.9 Hz, 3H, H3”). ^13^C NMR (100 MHz, CDCl_3_, *δ* = 77.0 ppm): *δ* 167.34 (C2), 157.48 (C4), 137.11 (C1a), 129.61 (C3a and C5a), 126.43 (C4a), 123.45 (C2a and C6a), 98.83 (C5), 82.03 (C1'), 80.99 (C3), 48.06 (C2'), 42.55 (C6'), 33.93 (C4'), 31.63 (C1”), 24.86 (C5'), 22.81 (C3'), 22.13 (C4”), 21.20 (C2”), 15.83 (C3”). m/z (%): 410 ([M + 2] 0,1), 408 ([M^+^.] 0,1), 207 (25), 172 (53), 191 (37), 173 (100), 144 (96), 95 (20), 83 (34), 69 (31), 41 (59).

#### 2.2.7 (S)‐3‐bromo‐4‐((4‐fluorophenyl)amino)‐5‐(((1R,2S,5R)‐2‐isopropyl‐5‐methylcyclohexyl)oxy)furan‐2(5H)‐one **5b**

White crystalline solid. TLC: Rf = 0.5 hexane/ethyl acetate (5:1 v/v). mp 149.6–150.6 °C. Yield: 67%. IR (ATR, $\bar{\nu }$/cm^−1^): 3177, 2951, 2869, 1729, 1636, 1203, 1133, 1049, 936, 505. ^1^H NMR (400 MHz, CDCl_3_, *δ* = 7.27 ppm): *δ* 7.22–7.16 (m, 2H, H2a and H6a), 7.10 (m, 2H, H3a and H5a), 6.01 (s, 1H, H5), 3.43 (td, *J =* 10.6, 4.5 Hz, 1H, H1'), 2.26–2.16 (m, 1H, H6'x), 1.68–1.54 (m, 2H, H3'x and H4'x), 1.37 (dtd, *J =* 14.5, 7.6, 7.2, 2.8 Hz, 2H, H1” and H5'), 1.18 (tdd, *J =* 12.6, 5.7, 2.7 Hz, 1H, H2'), 1.05 (td, *J =* 12.4, 10.8 Hz, 1H, H6'y), 0.91 (d, *J =* 6.6 Hz, 3H, H4”), 0.89–0.77 (m, 2H, H3'y and H4'y), 0.75 (d, *J =* 7.0 Hz, 3H, H2”), 0.52 (d, *J =* 7.0 Hz, 3H, H3”). ^13^C NMR (75 MHz, CDCl_3_, *δ* = 77.0 ppm): *δ* 167.30 (C2), 162.39 (C4a), 157.57 (C4), 132.95 (C1a), 126.31 (C6a), 126.22 (C2a), 116.48 (C5a), 116.25 (C3a), 98.75 (C5), 82.01 (C1'), 80.37 (C3), 48.05 (C2'), 42.59 (C6'), 33.90 (C4'), 31.64 (C5'), 24.97 (C1”), 22.80 (C3'), 22.11 (C4”), 21.15 (C2”), 15.85 (C3”). m/z (%): 428 ([M + 2] 1,16), 426 ([M^+^.] 1,16), 287 (30), 243 (21), 207 (21), 190 (34), 162 (22), 95 (39), 83 (84), 69 (54), 57 (46), 55 (100), 43 (61), 41 (79).

#### 2.2.8 (S)‐3‐bromo‐5‐(((1R,2S,5R)‐2‐isopropyl‐5‐methylcyclohexyl)oxy)‐4‐((4‐methoxyphenyl)amino)furan‐2(5H)‐one **6b**

White crystalline solid. TLC: Rf = 0.5 hexane/ethyl acetate (5:1 v/v). mp 142.9–143.9 °C. Yield: 78%. IR (ATR, $\bar{\nu }$/cm^−1^): 3166, 2951, 2869, 1729, 1636, 1516, 1234, 1121, 1039, 926, 546. ^1^H NMR (300 MHz, CDCl_3_, *δ* = 7.27 ppm): *δ* 7.15–7.09 (m, 2H, H2a and H6a), 6.91–6.86 (m, 2H, H3a and H5a), 5.97 (s, 1H, H5), 3.81 (s, 3H, H7a), 3.39 (td, *J =* 10.6, 4.5 Hz, 1H, H1'), 2.25–2.12 (m, 1H, H6'x), 1.57 (ddt, *J =* 12.5, 9.0, 2.6 Hz, 2H, H3'x and H4'x), 1.36–1.24 (m, 2H, H1” and H5'), 1.19–1.08 (m, 1H, H2'), 1.08–0.95 (m, 1H, H6'y), 0.88 (d, *J =* 6.5 Hz, 3H, H4”), 0.84–0.73 (m, 2H, H3'y and H4'y), 0.69 (d, *J =* 7.0 Hz, 3H, H2”), 0.47 (d, *J =* 6.9 Hz, 3H, H3”). ^13^C NMR (75 MHz, CDCl_3_, *δ* = 77.0 ppm): *δ* 167.46 (C2), 158.47 (C4), 158.23 (C4a), 129.66 (C1a), 126.20 (C2a and C6a), 114.57 (C3a and C5a), 98.67 (C5), 81.77 (C1'), 79.16 (C3), 55.47 (C7a), 48.03 (C2'), 42.63 (C6'), 33.91 (C4'), 31.63 (C5'), 24.75 (C1”), 22.76 (C3'), 22.08 (C4”), 21.20 (C2”), 15.88 (C3”). m/z (%): 440 ([M + 2] 0,03), 438 ([M^+^.] 0,03), 203 (100), 174 (78), 55 (31), 41 (29).

#### 2.2.9 (S)‐3‐bromo‐5‐(((1R,2S,5R)‐2‐isopropyl‐5‐methylcyclohexyl)oxy)‐4‐((3‐methoxyphenyl)amino)furan‐2(5H)‐one **7b**

White crystalline solid. TLC: Rf = 0.5 hexane/ethyl acetate (5:1 v/v). mp 148.8–149.8 °C. Yield: 82%. IR (ATR, $\bar{\nu }$/cm^−1^): 3218, 2941, 2869, 1738, 1637, 1596, 1491, 1134, 1039, 937, 752, 454. ^1^H NMR (400 MHz, CDCl_3_, *δ* = 7.27 ppm): *δ* 7.32–7.26 (m, 1H, H3a), 6.81 (dd, *J =* 7.9, 2.1 Hz, 1H, H4a), 6.74 (dd, *J =* 7.9, 2.1 Hz, 1H, H2a), 6.70 (s, 1H, H6a), 6.13 (s, 1H, H5), 3.84 (s, 3H, H7a), 3.42 (td, *J =* 10.6, 4.5 Hz, 1H, H1'), 2.27–2.20 (m, 1H, H6'x), 1.59 (ddt, *J =* 16.0, 13.0, 3.1 Hz, 2H, H3'x and H4'x), 1.38 (qtd, *J =* 11.5, 7.0, 3.8 Hz, 2H, H1” and H5”), 1.22–1.12 (m, 1H, H2'), 1.05 (d, *J =* 10.9 Hz, 1H, H6'y), 0.91 (d, *J =* 6.5 Hz, 3H, H4”), 0.88–0.76 (m, 2H, H3'y and H4'y), 0.71 (d, *J =* 7.0 Hz, 3H, H2”), 0.44 (d, *J =* 6.9 Hz, 3H, H3”). ^13^C NMR (100 MHz, CDCl_3_, *δ* = 77.0 ppm): *δ* 167.25 (C2), 160.61 (C5a), 157.43 (C4), 138.32 (C1a), 130.38 (C3a), 115.38 (C2a), 111.90 (C4a), 109.24 (C6a), 98.89 (C5), 82.13 (C1'), 81.40 (C3), 55.41 (C7a), 48.06 (C2'), 42.59 (C6'), 33.93 (C4'), 31.63 (C5'), 24.84 (C1”), 22.79 (C3'), 22.13 (C4”), 21.20 (C2”), 15.74 (C3”). m/z (%): 440 ([M + 2] 0,05), 438 ([M^+^.] 0,05), 203 (99), 174 (100), 83 (25), 67 (24), 55 (52), 41 (46).

### 2.3 Computational calculations

The three‐dimensional structures of two candidate diastereoisomers‐*RRSR* and *SRSR* relative configurations‐were built for each compound (**1–7**) using Wavefunction Spartan 14 (version 1.1.4).^54^ A conformational search was then carried out in the gas phase employing the MMFF force field and the Monte Carlo method, with a maximum of 1000 conformations per isomer. From these, the 10 lowest‐energy conformers with a Boltzmann population greater than 0.01% were selected for Density Functional Theory (DFT) calculations. Each selected conformer was subjected to geometry optimization and frequency analysis at the M06‐2X/6–31 + G(d,p) level of theory, as implemented in Gaussian 16^55^ (SI, Figs S65‐S71). Solvent effects were modeled using the polarizable continuum model (PCM), with chloroform as the solvent.

The files generated in this step were used to calculate the Nuclear Magnetic Resonance (NMR) shielding tensors at the B3LYP/6–311 + G(2d,p)^56^, ^57^, ^58^, ^59^, ^60^, ^61^, ^62^, ^63^, ^64^ level of theory using Gaussian 16. Shielding tensors for all nuclei were converted to referenced chemical shifts by subtracting the shielding tensors calculated for tetramethylsilane (TMS) at the same level of theory (^1^H = 31.89148; ^13^C = 183.3967). These calculations were performed for each optimized structure.

Theoretical ^13^C and ^1^H chemical shifts values obtained from DFT calculations were correlated with the experimental values through simple linear regression using the least squares method. Based on the regression‐adjusted values, the corrected mean absolute error (CMAE) was calculated as the average of the absolute differences between the corrected calculated (*δ*_corr calc_) and experimental (*δ*_exp_) chemical shifts, according to the equation, where *n* is the number of nuclei evaluated:

$$\text{CMAE}=\frac{\sum_{i=1}^{n}|\text{δcorr calc}-\delta \exp|}{n}$$

DP4 analysis was conducted using the online tool available at https://www-jmg.ch.cam.ac.uk/tools/nmr/DP4/. DP4+ analysis was performed using a Microsoft Excel® spreadsheet available at https://sarotti-nmr.weebly.com, where experimental and calculated chemical shift values were entered.

### 2.4 Herbicidal bioassays

The phytotoxic activity of the synthesized compounds **1** to **7** was selectively evaluated on monocotyledonous crops – forage sorghum (*Sorghum bicolor L. Moench*) and Marandu brachiaria (*Urochloa brizantha*) – and dicotyledonous crops – cucumber (*Cucumis sativus*), lettuce (*Lactuca sativa*), Santa Adélia Super tomato (*Solanum lycopersicum*), and beggartick (*Bidens pilosa*). These species exhibit a rapid response even at low concentrations of active ingredients. The experiment was conducted in 90 mm diameter Petri dishes containing germination paper, with 20 seeds of each species per dish and three replicates.

Five milliliters of the prepared solutions were added to each dish. After labeling and sealing with plastic film, the dishes were incubated in a germination chamber (Biological Oxygen Demand Incubator – B.O.D.) at 25 °C in the dark for 120 h (5 days). Following incubation, the dishes were removed and stored at 0 °C for 24 h to halt growth, facilitating seedling handling in the subsequent stage. Seedlings were arranged on a rubber plate with a black background and photographed. The lengths of the shoot and radicle were measured using ImageJ® software.

Solutions of each compound (**1**–**7**) were prepared at five concentrations: 50, 75, 150, 300, and 500 μM. The compounds were initially dissolved in a 2.0% (m/v) gum arabic solution to prepare the maximum concentration (500 μM). Lower concentrations were obtained by diluting this stock solution with the same gum arabic concentration.

The commercial herbicide Dual® (Dual Gold, Syngenta® Company, São Paulo, Brazil) served as the positive control. Its active ingredient, *S*‐metolachlor, was solubilized in 2.0% (m/v) gum arabic at the same concentrations tested. An aqueous solution of 2.0% (m/v) gum arabic was used as the negative control.

Statistical analyses were conducted using Microsoft Excel® software. The data, expressed as percentage of shoots or roots growth inhibition with respect to untreated controls, were analyzed using Tukey's test at 0.05 probability level.

The percentage of growth, *G(%)*, of shoots and roots was calculated according to the following formula:

$$G\left(\%\right)=\frac{\left(S-C\right)}{C}\;x\;100$$

where *S* is the average length of the germinating shoots or roots of the plants, and *C* corresponds to the average growth of the negative control.

## 3 RESULTS AND DISCUSSION

### 3.1 Synthesis

Mucobromic acid is an oxo acid that exists in equilibrium between its cyclic and acyclic forms. The acyclic form is activated by protonation of the carbonyl group, a process that only occurs under acidic conditions. (1*S*)‐(+)‐10‐Camphorsulfonic acid (CSA) is used as the acid catalyst in this reaction. Upon activation, the carbonyl group becomes more electrophilic. The oxygen atom of the alcohol's hydroxyl group then attacks the carbon of the activated carboxylic group, forming a tetrahedral intermediate. This intermediate subsequently collapses, resulting in the elimination of a water molecule. The acid catalyst is regenerated, and under the reaction conditions employed, butenolide **1b** is formed (Scheme 2).

**Scheme 2:**
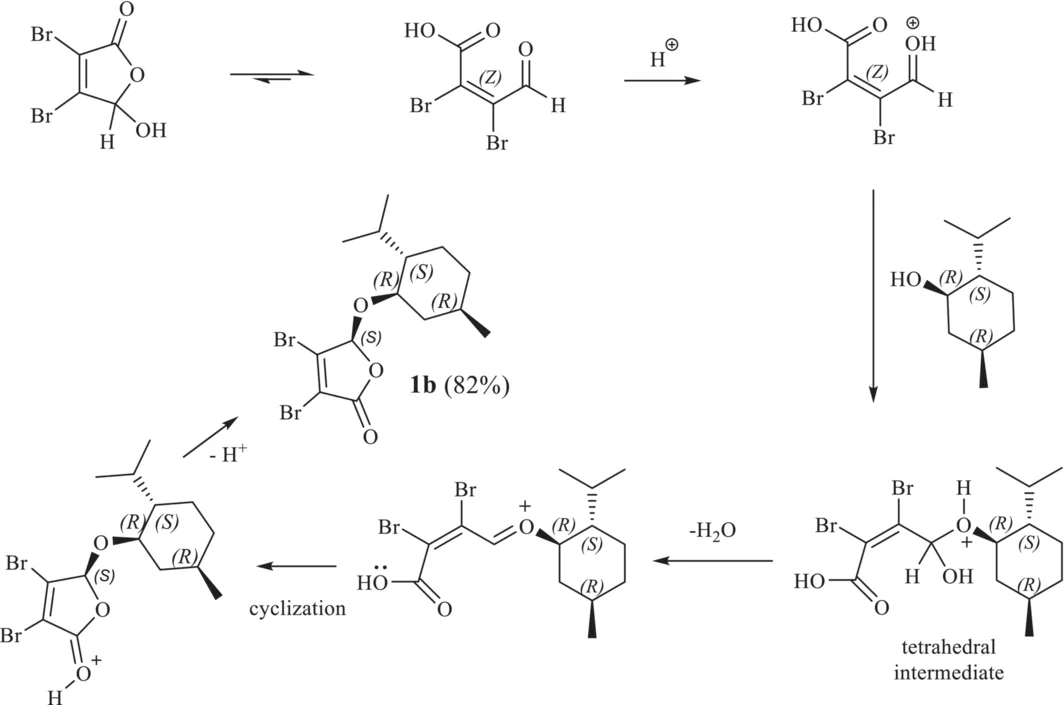
Synthesis of (*R*)‐3,4‐dibromo‐5‐(((1*R*,2*S*,5*R*)‐2‐isopropyl‐5‐methylcyclohexyl)oxy)furan‐2(5*H*)‐one (**1b**).

The synthesis of the new tetronamides from butenolide **1b**, as outlined in Scheme 3, involves an aza‐Michael addition/elimination reaction on the butenolide ring. Different substituted anilines were used as the nucleophilic species, reacting with the activated C4 carbon of butenolide **1b**, resulting in the formation of tetronamides **2** through **7**.

**Scheme 3:**
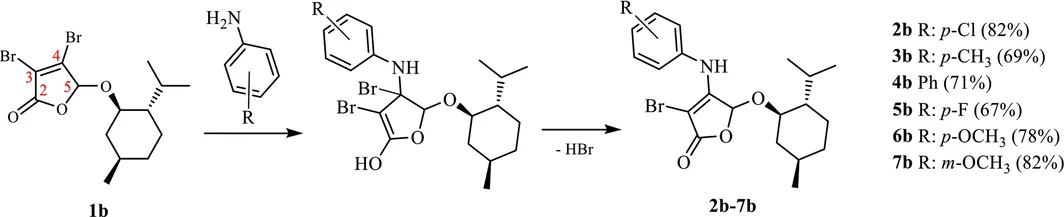
Synthetic route for obtaining new tetronamides **2b**‐**7b** and their yields.

### 3.2 Structural identification

Compounds **2b**–**7b** are novel and exhibit structural similarities. Therefore, only the spectroscopic analysis of tetronamide **2b**, *SRSR* isomer, is presented in detail as representative of the series. Spectrometric data for all synthesized compounds are provided in the *Materials and Methods* section, and the corresponding spectra are available in the Supplementary Information (SI, Figs S1‐S63).

The IR spectrum of **2b** (SI, Fig. S10) showed characteristic absorptions at 3177 cm^−1^ (N—H), 2957–2871 cm^−1^ (aliphatic C—H), 1719 cm^−1^ (C=O), 1634 cm^−1^ (C=C), 1339 cm^−1^ (C—N), 1135 cm^−1^ (C–O–C), and 590 cm^−1^ (C—Cl), consistent with the proposed structure. In the EI mass spectrum (70 eV) (SI, Fig. S11), the molecular ion was detected as a weak peak at *m/z* 443, reflecting the high fragmentation tendency of **2b**. The base peak at *m/z* 207, together with the isotope peak at *m/z* 209 (^37^Cl), confirmed the presence of chlorine in the major fragment.

To elucidate the structural configuration of compound **2b**, a detailed analysis of the ^1^H and ^13^C NMR spectra was conducted (SI, Figs S12 and S13), complemented by DEPT (SI, Fig. S14), COSY (SI, Fig. S15), NOESY (SI, Fig. S16), HSQC (SI, Fig. S17), and HMBC (SI, Fig. S18) experiments. In the ^1^H NMR spectrum, two multiplets were observed at *δ* = 7.37 ppm and *δ* = 7.13 ppm, attributed to hydrogens H3a/H5a and H2a/H6a, respectively. The doublet at *δ* = 0.91 ppm (*J =* 6 Hz) was assigned to the methyl hydrogens of the methyl group H4”. The multiplet between *δ* = 0.87 and 0.78 ppm was attributed to hydrogens H3'y and H4'y, confirmed by HSQC correlations to their respective carbons. Two doublets at *δ* = 0.75 and *δ* = 0.50 ppm (*J =* 6 Hz) were assigned to the methyl hydrogens of the isopropyl group H2” and H3”, respectively.

In the ^13^C NMR spectrum, significant signals were detected at *δ* = 135.66, 132.10, 129.69, and 124.93 ppm, and assigned to the aromatic carbons C1a, C4a, C3a/C5a, and C2a/C6a, respectively. The most deshielded signal was observed at *δ* = 167.13 ppm and assigned to carbon C2, corresponding to the carbonyl group. The second most deshielded signal, at *δ* = 157.11 ppm, was attributed to the olefinic carbon C4. The signal at *δ* = 98.80 ppm was attributed to carbon C5, while the signal at *δ* = 82.15 ppm corresponded to the olefinic carbon C1'. Carbons C3', C4', and C6' showed signals at *δ* = 22.80, 33.89, and 42.59 ppm, respectively. In the DEPT‐135 spectrum, these signals appeared with phase inversion, a typical feature of methylene (CH_2_) groups. The signal at *δ* = 48.04 ppm was assigned to carbon C2'. Additionally, the signals at *δ* = 42.59, 33.89, and 31.63 ppm were assigned to carbons C6', C4', and C5', respectively. The signal at *δ* = 24.98 ppm was attributed to carbon C1”. Signals at *δ* = 22.12, 21.12, and 15.83 ppm were assigned to the methyl carbons C4”, C2”, and C3”, respectively. Together, these spectroscopic data confirm the signal assignments and support the proposed chemical structure of tetronamide **2b**.

### 3.3 Computational calculations

The seven synthesized tetronamides (**1**–**7**), each considering the stereoisomeric configurations *RRSR* and *SRSR*, were evaluated through theoretical calculations based on Density Functional Theory (DFT). For the sake of simplicity and clarity in presenting the results, the *RRSR* configuration was designated as ‘**a**’ and the *SRSR* configuration as ‘**b**,’ resulting in the denominations **1a**, **2a**, **3a**, **4a**, **5a**, **6a**, **7a** and **1b**, **2b**, **3b**, **4b**, **5b**, **6b**, **7b**, respectively (Fig. 1 and SI, Fig. S64).

**Figure 1:**
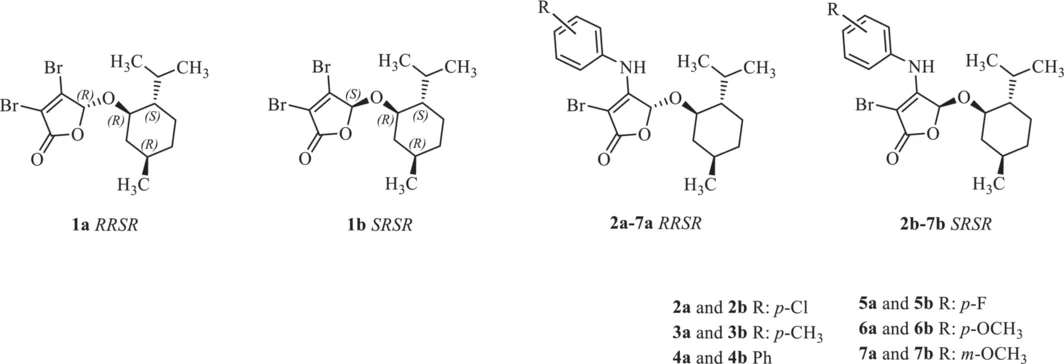
Molecular structures of tetronamides **1**–**7** showing the relative configurations *RRSR* (**a**) and *SRSR* (**b**).

Using these designations, geometry optimization and frequency calculations were performed at the M06‐2X/6–31 + G(d,p) level of theory, followed by ^13^C and ^1^H chemical shifts calculations employing the B3LYP/6–311 + G(2d,p) level (SI, Figs S65‐S71). The theoretical values obtained were correlated with experimental chemical shifts via linear regression (SI, Figs S72‐S99), with the quality of the fit evaluated by the coefficient of determination (R^2^), the corrected mean absolute error (CMAE), and the probabilistic analyses DP4 and DP4+ (SI, Figs S100‐S113).^38^, ^40^, ^41^

The CMAE corresponds to the mean of the absolute differences between the calculated chemical shifts (*δ*_corr calc_), corrected by linear regression, and the experimental chemical shifts (*δ*_exp_) for each nucleus. The DP4 method utilizes an advanced statistical framework to assess the compatibility between theoretical and experimental NMR data and is traditionally applied when experimental data are available for a single compound. DP4+ is an enhanced version that assigns experimental data to multiple candidate structures, calculating the probability of each matching the actual structure by considering both scaled and unscaled chemical shifts.

The comparison between calculated and experimental chemical shifts revealed consistent trends, allowing differentiation of the *RRSR* and *SRSR* isomers. Tetronamides **1b**, **2b**, **3b**, **4b**, **5b**, **6b**, and **7b** showed the lowest CMAE values; and DP4 and DP4+ probabilities of 100%, unequivocally confirming the *SRSR* configuration. Linear regression was also conclusive, with R^2^ values greater than those observed for the *RRSR* isomers (Table 1).

**Table 1:** Results of DP4, DP4+, R^2^ and CMAE probabilistic analysis

Comp.	DP4 (%)			DP4+ (%)			R^2^		CMAE (ppm)	
	^13^C	^1^H	^13^C and ^1^H	^13^C	^1^H	^13^C and ^1^H	^13^C	^1^H	^13^C	^1^H
1a	0	0	0	100	100	100	0.982	0.924	5.61	0.34
1b	**100**	**100**	**100**	**100**	**100**	**100**	**0.990**	**0.999**	**3.75**	**0.06**
2a	0	0	0	0	0	0	0.995	0.989	2.84	0.18
2b	**100**	**100**	**100**	**100**	**100**	**100**	**0.995**	**0.998**	**2.42**	**0.07**
3a	0	0	0	0	0	0	0.996	0.979	2.51	0.24
3b	**100**	**100**	**100**	**100**	**100**	**100**	**0.998**	**0.996**	**1.65**	**0.10**
4a	1.2	0	0	0	0	0	0.995	0.984	2.78	0.24
4b	**98.8**	**100**	**100**	**100**	**100**	**100**	**0.996**	**0.997**	**2.41**	**0.09**
5a	0	0	0	0	0	0	0.997	0.987	2.27	0.20
5b	**100**	**100**	**100**	**100**	**100**	**100**	**0.998**	**0.996**	**1.49**	**0.09**
6a	0	0.4	0	0	0	0	0.995	0.990	2.70	0.18
6b	**100**	**99.6**	**100**	**100**	**100**	**100**	**0.997**	**0.997**	**2.00**	**0.09**
7a	0.1	0	0	0	0	0	0.998	0.985	2.13	0.19
7b	**99.9**	**100**	**100**	**100**	**100**	**100**	**0.998**	**0.999**	**1.72**	**0.07**

The experimental and calculated ^13^C and ^1^H NMR spectra of tetronamide **2** were processed and compiled using the MestreNova software and are shown in Figs 2 and 3. A high degree of agreement between the spectral profiles is observed, clearly demonstrating the similarity between the theoretical and experimental data. The average energies of the optimized structures of the conformers of tetronamides **1**–**7** are presented in Table 2. The results uniformly indicate lower energy for the *RRSR* isomers compared to their corresponding *SRSR* isomers, suggesting that *RRSR* is the thermodynamic product while *SRSR* is the kinetic product. However, the energy differences between the two isomers of each tetronamide are small, ranging from 0.49 to 1.07 kcal mol^−1^.

**Figure 2:**
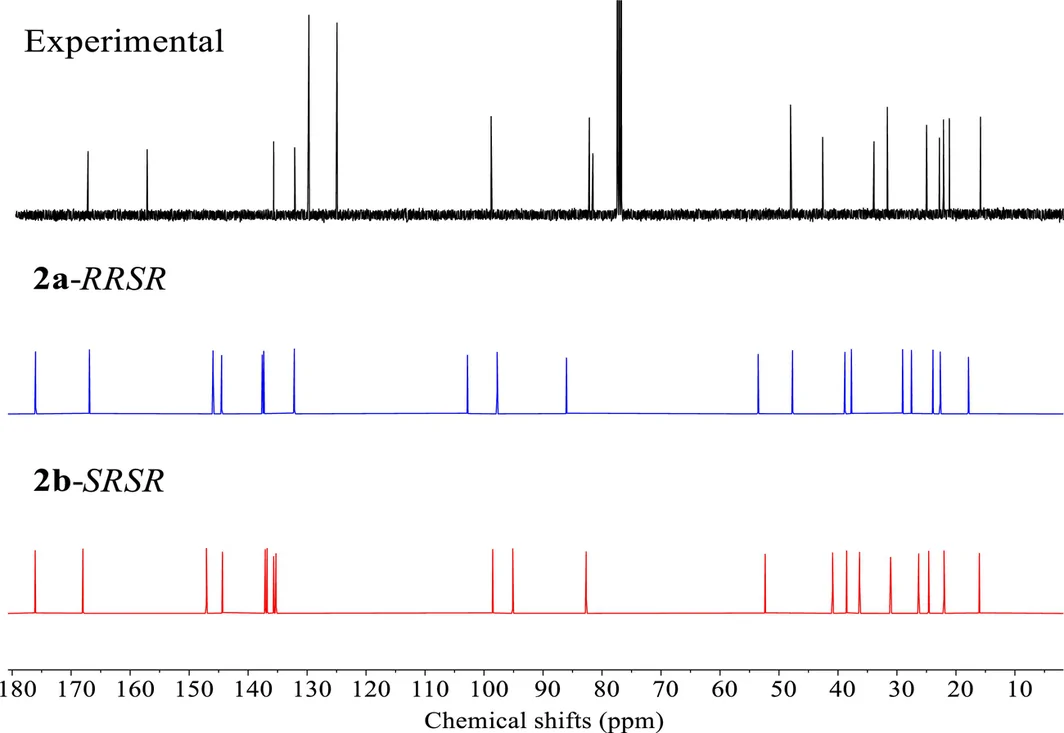
Experimental and theoretical ^13^C NMR spectra of compound **2**. The theoretical spectrum (**2a‐** *RRSR* and **2b‐** *SRSR*) was calculated at B3LYP/6–311 + G(2d,p)//M06‐2X/6–31 + G(d,p) level of theory. The experimental and calculated spectra were assembled in MestreNova.

**Figure 3:**
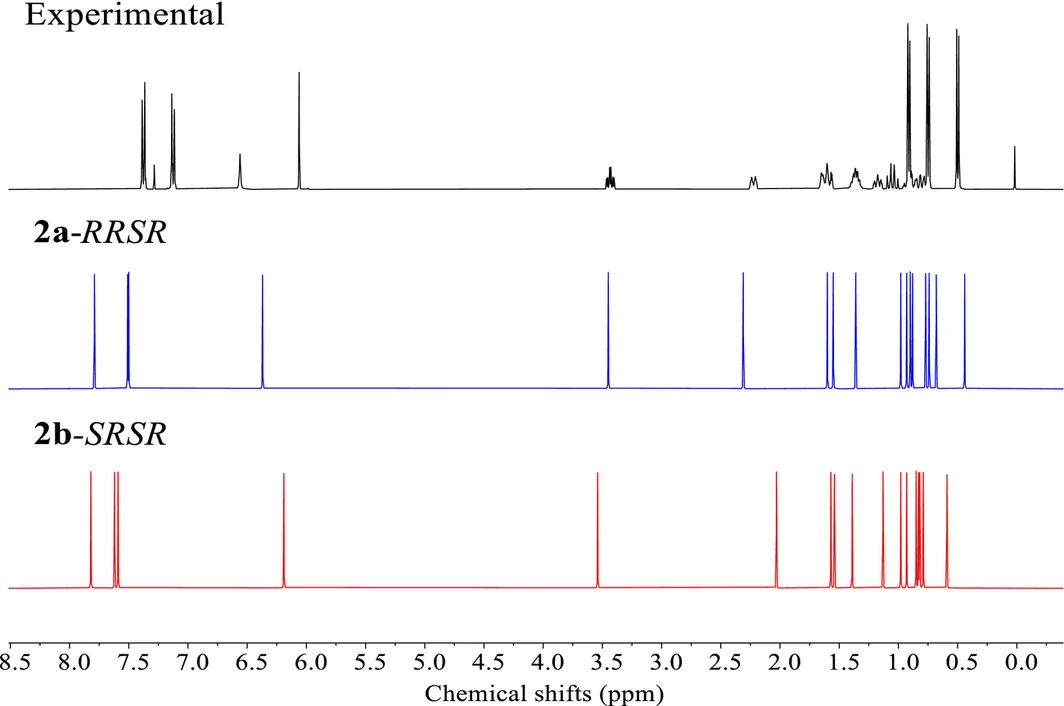
Experimental and theoretical ^1^H NMR spectra of compound **2**. The theoretical spectrum (**2a**‐*RRSR* and **2b**‐*SRSR*) was calculated at B3LYP/6–311 + G(2d,p)//M06‐2X/6–31 + G(d,p) level of theory. The experimental and calculated spectra were assembled in MestreNova.

**Table 2:** Average energy values of the optimized structures of each compound

Tetronamide	Isomer	Energy (kcal mol^−1^)	Energy (kJ mol^−1^)	Energy (Hartree)
1a	*RRSR*	−3 711 246.73920	−15 527 856.3568	−5914.24892
1b	*SRSR*	−3 711 252.81656	−15 527 881.7845	−5914.25861
2a	*RRSR*	−2 565 794.81098	−10 735 285.4892	−4088.85484
2b	*SRSR*	−2 565 794.13650	−10 735 282.6671	−4088.85377
3a	*RRSR*	−2 302 049.33790	−9 631 774.4298	−3668.54962
3b	*SRSR*	−2 302 048.76843	−9 631 772.0471	−3668.54871
4a	*RRSR*	−2 277 404.19641	−9 528 659.1578	−3629.27508
4b	*SRSR*	−2 277 403.66560	−9 528 656.9369	−3629.27424
5a	*RRSR*	−2 339 666.46515	−9 789 164.4902	−3728.49634
5b	*SRSR*	−2 339 665.39054	−9 789 159.9940	−3728.49462
6a	*RRSR*	−2 349 225.09563	−9 829 157.8001	−3743.72899
6b	*SRSR*	−2 349 224.60360	−9 829 155.7415	−3743.72820
7a	*RRSR*	−2 349 229.64209	−9 829 176.8225	−3743.73623
7b	*SRSR*	−2 349 229.15485	−9 829 174.7839	−3743.73545

The structures of the conformers were optimized at M06‐2X/6–31 + G(d,p) level of theory.

### 3.4 Herbicidal bioassays

The results of the bioassays were presented in bar graphs with their respective standard deviations. The values of inhibition and stimulation of the substances were evaluated according to the growth (positive values) or inhibition (negative values) of the root and stem of the tested seeds. The commercial herbicide Dual Gold® (Syngenta® Company, São Paulo, Brazil; *S*‐metolachlor) was used as positive control and an aqueous solution of 2.0% (m/v) gum arabic was used as the negative control.

#### 3.4.1 Cucumis sativus

The results presented (SI, Figs S114 and S115, Tables S1 and S2) indicate that compounds **1**–**7** inhibited the development of radicles and shoots in cucumber seedlings during the phytotoxicity assay, although to a lesser extent than the commercial herbicide. Compound **1b** inhibited shoot growth by 46.57% at a concentration of 150 μM and radicle growth by 67.37% at 300 μM. Some compounds stimulated radicle growth, such as compound **2b** at 50 μM and compound **7b** at both 50 μM and 300 μM.

#### 3.4.2 Solanum lycopersicum

The tests performed with tomato seedlings mostly indicated stimulation of rootlet and shoot growth (SI, Figs S116 and S117, Tables S3 and S4). Only compound **1b** at 50 μM and compound **7b** at 150 μM, 250 μM, and 500 μM showed inhibition of rootlet growth, which was much lower than that caused by the commercial herbicide. Compound **7b** also exhibited slight inhibition of shoot growth at 150 μM and 500 μM.

#### 3.4.3 *Sorghum bicolor* L. Moench

Bioassays with sorghum demonstrated that most compounds stimulated root and shoot growth (SI, Figs. S118 and S119, Tables S5 and S6). The greatest stimulation occurred at 1000 μM of compound **2b**, which promoted the most significant increase in root and shoot growth. Consistent with the results observed in previous species, the commercial herbicide caused greater growth inhibition at all tested concentrations.

#### 3.4.4 Urochloa brizantha

The brachiaria assays indicated a significant reduction in root and shoot growth for most of the compounds tested (Fig. 4, and SI, Tables S7 and S8). Compounds **1b**, **3b**, and **4b** stood out, exhibiting root inhibition greater than 60% at a concentration of 300 μM. Compound **1b**, at 75 μM, caused 55.9% inhibition, a value higher than that observed for the commercial herbicide. In the shoot assays, compounds **1b** and **5b** demonstrated inhibition exceeding 60% at 300 μM.

**Figure 4:**
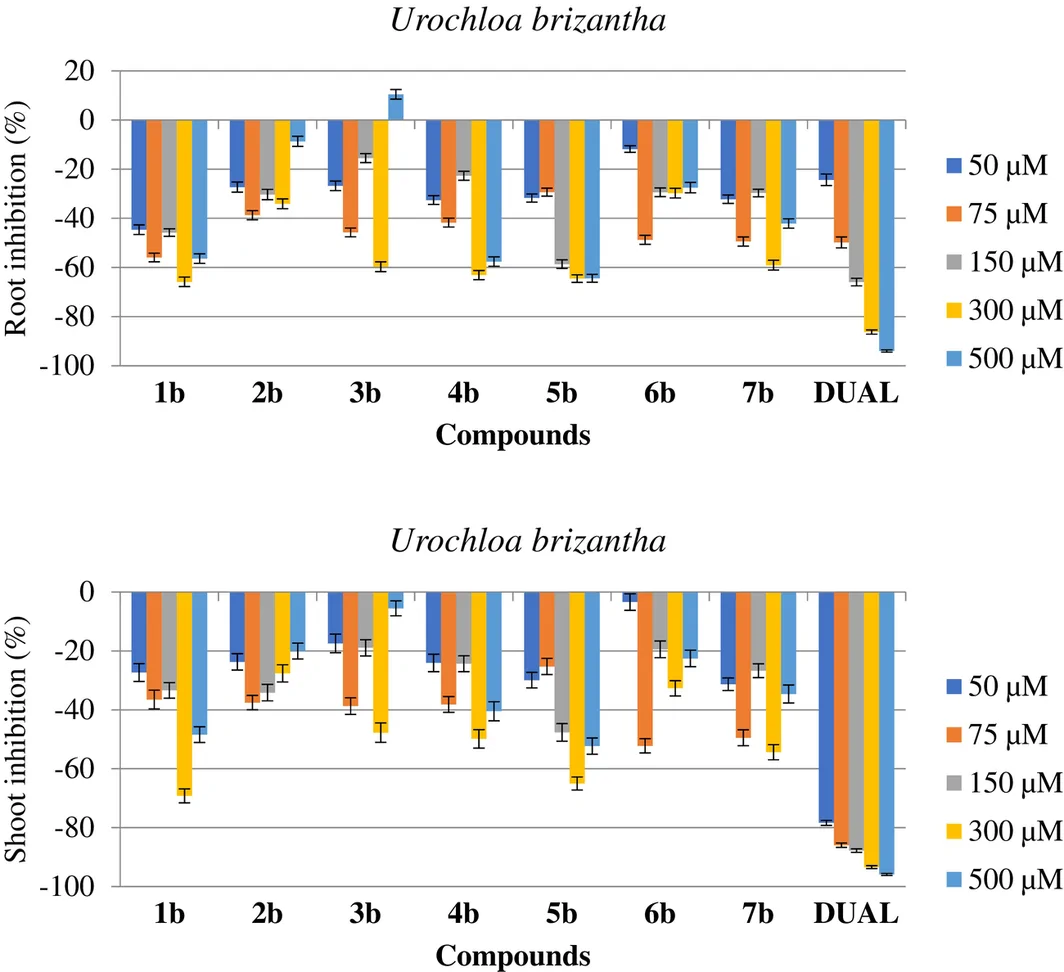
Development of the roots and shoots of brachiaria.

#### 3.4.5 Bidens pilosa

Bioassays with beggartick showed that most compounds inhibited rootlet and shoot growth, but no statistically significant differences were observed between treatment groups, suggesting a similar overall response across different concentrations (SI, Figs S120 and S121, Tables S9 and S10).

Unlike the commercial herbicide, the synthesized tetronamides did not exhibit a clear concentration‐dependent response. For several compounds, the highest phytotoxic effects were observed at intermediate concentrations, whereas increasing the concentration did not consistently enhance inhibition. This behavior suggests that phytotoxicity is not governed solely by the applied concentration within the evaluated range.

Several factors may contribute to this response. The relatively hydrophobic nature of the menthol‐derived tetronamides may limit their aqueous availability or uptake by plant tissues at higher concentrations. Although these hypotheses require further investigation, they are consistent with the absence of a classical dose–response relationship.

Despite the absence of a consistent dose–response relationship, several compounds produced substantial inhibition of *Urochloa brizantha* root and shoot growth, with inhibition values exceeding 60% for some derivatives. These results indicate that the tetronamide scaffold possesses intrinsic phytotoxic activity, although further structural optimization will be necessary to improve potency and achieve more predictable concentration‐dependent responses.

Collectively, the results reveal a marked species‐dependent phytotoxic profile. Whereas cucumber, tomato and sorghum were only weakly affected or even exhibited growth stimulation, *Urochloa brizantha* showed substantial susceptibility to several tetronamide derivatives. This differential response suggests a degree of selectivity that may be advantageous for herbicide discovery.

## 4 CONCLUSIONS

Six novel tetronamides derived from mucobromic acid and menthol were synthesized in significant yields, and their structures were rigorously characterized using advanced spectroscopic techniques, supported by theoretical DFT calculations and statistical analyses including DP4, DP4+, linear regression (R^2^), and CMAE. The *SRSR* stereochemical configurations of tetronamides **1**–**7** were unequivocally determined, confirming the accuracy of the structural characterization aided by computational methods. Moreover, all compounds exhibited significant effects on seed development in phytotoxicity assays, with inhibition observed in relevant target species such as *U. brizantha*. These findings offer new prospects for the development of selective bioactive agents with potential applications in sustainable agriculture and weed management, establishing tetronamides as promising chemical tools for future weed control.

## CONFLICT OF INTEREST

The authors declare no conflict of interest.

## Supporting information


**Data S1.** Supporting Information.

## Data Availability

The data that supports the findings of this study are available in the supplementary material of this article.
