# Supplementary material for: Novel tetronamides derived from mucobromic acid: synthesis, structural elucidation, DFT calculations and phytotoxic activity
Source: Pest Manag Sci. 2026 Aug 6;82(12):11853–63. doi: 10.1002/ps.71179 (PMC13756821; doi:10.1002/ps.71179)
Supplement: Supplementary file 1 [file PS-82-11853-s001.docx]

**SUPPLEMENTARY INFORMATION**

**Novel Tetronamides Derived from Mucobromic Acid: Synthesis, Structural Elucidation, DFT calculations and Phytotoxic Activity**

**Kamylla C. F. de Faria, Vitor C. Baia, Elson S. Alvarenga***

Department of Chemistry, Universidade Federal de Viçosa, 36570-900, Viçosa, MG, Brazil

*****Corresponding author: [elson@ufv.br](mailto:elson@ufv.br)

**Figure S1.** IR spectrum of compound **1b**.

**Figure S2.** Mass spectrum of compound **1b**.

**Figure S3.** ^1^H NMR spectrum (300 MHz, CDCl_3;_ δ 7.26 ppm) of compound **1b**.

**Figure S4.** ^13^C NMR spectrum (100 MHz, CDCl_3;_ δ 77.0 ppm) of compound **1b**.

**Figure S5.** DEPT spectrum (100 MHz, CDCl_3_) of compound **1b**.

**Figure S6.** COSY contour map of compound **1b** (400 MHz, CDCl_3_).

**Figure S7.** NOESY contour map of compound **1b** (400 MHz, CDCl_3_)..

**Figure S8.** HSQC contour map of compound **1b** (400 MHz, CDCl_3_).).

**Figure S9.** HMBC contour map of compound **1b** (400 MHz, CDCl_3_).).

**Figure S10.** IR spectrum of tetronamide **2b**.

**Figure S11.** Mass spectrum of compound **2b**.

**Figure S12.** ^1^H NMR spectrum (400 MHz, CDCl_3;_ *δ=* 7.26 ppm) of compound **2b**.

**Figure S13.** ^13^C NMR spectrum (100 MHz, CDCl_3_, *δ* = 77.0 ppm) of compound **2b**.

**Figure S14.** DEPT-135 spectrum (100 MHz, CDCl_3_ *δ* = 77.0 ppm) of compound **2b**.

**Figure S15.** COSY contour map of compound **2b** (400 MHz, CDCl_3_).

**Figure S16.** NOESY contour map of compound **2b** (400 MHz, CDCl_3_).

**Figure S17.** HSQC contour map of compound **2b** (400 MHz, CDCl_3_).

**Figure S18.** HMBC contour map of compound **2b** (400 MHz, CDCl_3_).

**Figure S19.** IR spectrum of compound **3b**.

**Figure S20.** Mass spectrum of compound **3b**.

**Figure S21.** ^1^H NMR spectrum (400 MHz, CDCl_3;_ δ 7.26 ppm) of compound **3b**.

**Figure S22.** ^13^C NMR spectrum (75 MHz, CDCl_3;_ δ 77.0 ppm) of compound **3b**.

**Figure S23.** DEPT spectrum (75 MHz) of compound **3b**.

**Figure S24.** COSY contour map of compound **3b** (300 MHz, CDCl_3_).

**Figure S25.** NOESY contour map of compound **3b** (300 MHz, CDCl_3_).

**Figure S26.** HSQC contour map of compound **3b** (400 MHz, CDCl_3_).

**Figure S27.** HMBC contour map of compound **3b** (400 MHz, CDCl_3_).

**Figure S28.** IR spectrum of compound **4b**.

**Figure S29.** Mass spectrum of compound **4b**.

**Figure S30.** ^1^H NMR spectrum (400 MHz, CDCl_3;_ δ 7.26 ppm) of compound **4b**.

**Figure S31.** ^13^C NMR spectrum (100 MHz, CDCl_3;_ δ 77.0 ppm) of compound **4b**.

**Figure S32.** DEPT spectrum of compound **4b** (100 MHz, CDCl_3_).

**Figure S33.** COSY contour map of compound **4b** (400 MHz, CDCl_3_)

**Figure S34.** NOESY contour map of compound **4b** (400 MHz, CDCl_3_).

**Figure S35.** HSQC contour map of compound **4b** (400 MHz, CDCl_3_).

**Figure S36.** HMBC contour map of compound **4b** (400 MHz, CDCl_3_).

**Figure S37.** IR spectrum of compound **5b**.

**Figure S38.** Mass spectrum of compound **5b**.

**Figure S39.** ^1^H NMR spectrum (400 MHz, CDCl_3;_ δ 7.26 ppm) of compound **5b**.

**Figure S40.**^13^C NMR spectrum (75 MHz, CDCl_3;_ δ 77.0 ppm) of compound **5b**.

**Figure S41.** DEPT spectrum (75 MHz) of compound **5b**.

**Figure S42.** COSY contour map of compound **5b** (400 MHz, CDCl_3_).

**Figure S43.** NOESY contour map of compound **5b** (400 MHz, CDCl_3_).

**Figure S44.** HSQC contour map of compound **5b** (400 MHz, CDCl_3_).

**Figure S45.** HMBC contour map of compound **5b** (400 MHz, CDCl_3_).

**Figure S46.** IR spectrum of compound **6b**.

**Figure S47.** Mass spectrum of compound **6b**.

**Figure S48.** ^1^H NMR spectrum (300 MHz, CDCl_3;_ δ 7.26 ppm) of compound **6b**.

**Figure S49.** ^13^C NMR spectrum (75 MHz, CDCl_3;_ δ 77.0 ppm) of compound **6b**.

 **Figure S50.** DEPT spectrum (75 MHz*,* CDCl_3_) of compound **6b**.

**Figure S51.** COSY contour map of compound **6b** (300 MHz, CDCl_3_).

**Figure S52.** NOESY contour map of compound **6b** (400 MHz, CDCl_3_).

**Figure S53.** HSQC contour map of compound **6b** (400 MHz, CDCl_3_).

**Figure S54.** HMBC contour map of compound **6b** (400 MHz, CDCl_3_).

**Figure S55.** IR spectrum of compound **7b**.

**Figure S56.** Mass spectrum of compound **7b**.

**Figure S57.** ^1^H NMR spectrum (400 MHz; CDCl_3_; δ 7.26 ppm) of compound **7b**.

**Figure S58.** ^13^C NMR spectrum (100 MHz; DMSO-d_6_; δ 40.0 ppm) of compound **7b**.

**Figure S59.** DEPT spectrum (100 MHz, CDCl_3_) of compound **7b**.

**Figure S60.** COSY contour map of compound **7b** (400 MHz, CDCl_3_).

**Figure S61.** NOESY contour map of compound **7b** (400 MHz, CDCl_3_).

**Figure S62.** HSQC contour map of compound **7b** (400 MHz, CDCl_3_).

**Figure S63.** HMBC contour map of compound **7b** (400 MHz, CDCl_3_).

**Figure S64**. Molecular structures of tetronamides **1**–**7** showing the relative configurations *RRSR* (**a**) and *SRSR* (**b**).

**Figure S65.** Three-dimensional structure of the most stable conformer of tetronamide **1**, with geometry optimized using Gaussian 16 at the M06-2X/6-31+G(d,p) level of theory.

**Figure S66.** Three-dimensional structure of the most stable conformer of tetronamide **2**, with geometry optimized using Gaussian 16 at the M06-2X/6-31+G(d,p) level of theory.

**Figure S67.** Three-dimensional structure of the most stable conformer of tetronamide **3**, with geometry optimized using Gaussian 16 at the M06-2X/6-31+G(d,p) level of theory.

**Figure S68.** Three-dimensional structure of the most stable conformer of tetronamide **4**, with geometry optimized using Gaussian 16 at the M06-2X/6-31+G(d,p) level of theory.

**Figure S69.** Three-dimensional structure of the most stable conformer of tetronamide **5**, with geometry optimized using Gaussian 16 at the M06-2X/6-31+G(d,p) level of theory.

**Figure S70.** Three-dimensional structure of the most stable conformer of tetronamide **6**, with geometry optimized using Gaussian 16 at the M06-2X/6-31+G(d,p) level of theory.

**Figure S71.** Three-dimensional structure of the most stable conformer of tetronamide **7**, with geometry optimized using Gaussian 16 at the M06-2X/6-31+G(d,p) level of theory.

Carbon - RRSR

Calc

| Exp | RRSR |
| --- | --- |
| 164.25 | 172.923 |
| 143.44 | 171.166 |
| 118.48 | 140.25 |
| 104.47 | 106.431 |
| 84.46 | 87.155 |
| 48.03 | 51.3572 |
| 42.15 | 33.3466 |
| 33.96 | 32.5711 |
| 31.62 | 32.6284 |
| 25.26 | 36.6205 |
| 22.89 | 27.5342 |
| 22.07 | 25.9928 |
| 20.86 | 21.1937 |
| 15.86 | 20.0247 |

**Figure S72.** Correlation between the experimental ^13^C NMR chemical shifts (x-axis) and the calculated values (y-axis) for compound **1**, RRSR isomer. The linear regression line is represented by the equation shown in the plot, along with the coefficient of determination (R^2^). The chemical shifts were calculated at the B3LYP/6-311+G(2d,p)//M06-2X/6-31+G(d,p) level of theory using Gaussian 16.

Carbon - SRSR

Calc

| Exp | SRSR |
| --- | --- |
| 164.25 | 172.862 |
| 143.44 | 170.772 |
| 118.48 | 140.179 |
| 104.47 | 109.017 |
| 84.46 | 89.2535 |
| 48.03 | 53.3935 |
| 42.15 | 46.641 |
| 33.96 | 38.9063 |
| 31.62 | 37.9926 |
| 25.26 | 32.0601 |
| 22.89 | 28.0525 |
| 22.07 | 23.8461 |
| 20.86 | 21.5442 |
| 15.86 | 18.4328 |

**Figure S73.** Correlation between the experimental ^13^C NMR chemical shifts (x-axis) and the calculated values (y-axis) for compound **1**, SRSR isomer. The linear regression line is represented by the equation shown in the plot, along with the coefficient of determination (R^2^). The chemical shifts were calculated at the B3LYP/6-311+G(2d,p)//M06-2X/6-31+G(d,p) level of theory using Gaussian 16.

Hydrogen - RRSR

Calc

| Exp | RRSR |
| --- | --- |
| 5.81 | 6.16499 |
| 3.58 | 4.2203 |
| 2.32 | 1.58622 |
| 2.24 | 1.7859 |
| 1.42 | 1.87557 |
| 1.68 | 1.58288 |
| 1.11 | 1.79439 |
| 0.86 | 1.24018 |
| 1.38 | 1.86325 |
| 1.02 | 1.21391 |
| 1.66 | 1.72934 |
| 0.94 | 1.14149 |
| 0.92 | 0.905721 |
| 0.82 | 0.938793 |

**Figure S74.** Correlation between the experimental ^1^H NMR chemical shifts (x-axis) and the calculated values (y-axis) for compound **1**, RRSR isomer. The linear regression line is represented by the equation shown in the plot, along with the coefficient of determination (R^2^). The chemical shifts were calculated at the B3LYP/6-311+G(2d,p)//M06-2X/6-31+G(d,p) level of theory using Gaussian 16.

Hydrogen - SRSR

Calc

| Exp | SRSR |
| --- | --- |
| 5.81 | 6.01092 |
| 3.58 | 3.75363 |
| 2.32 | 2.36553 |
| 2.24 | 2.25658 |
| 1.42 | 1.45399 |
| 1.68 | 1.70795 |
| 1.11 | 1.10902 |
| 0.86 | 0.878686 |
| 1.38 | 1.40362 |
| 1.02 | 1.12879 |
| 1.66 | 1.6921 |
| 0.94 | 0.966225 |
| 0.92 | 0.9234 |
| 0.82 | 0.989537 |

**Figure S75.** Correlation between the experimental ^1^H NMR chemical shifts (x-axis) and the calculated values (y-axis) for compound **1**, SRSR isomer. The linear regression line is represented by the equation shown in the plot, along with the coefficient of determination (R^2^). The chemical shifts were calculated at the B3LYP/6-311+G(2d,p)//M06-2X/6-31+G(d,p) level of theory using Gaussian 16.

Carbon - RRSR

Calc

| Exp | RRSR |
| --- | --- |
| 167.13 | 176.441 |
| 157.11 | 168.001 |
| 135.66 | 144.345 |
| 132.1 | 147.071 |
| 129.69 | 136.779 |
| 129.69 | 137.087 |
| 124.93 | 135.275 |
| 124.93 | 135.663 |
| 98.8 | 98.5365 |
| 81.54 | 95.1277 |
| 82.15 | 82.6817 |
| 48.04 | 52.3391 |
| 42.59 | 40.9219 |
| 33.89 | 38.5256 |
| 31.63 | 31.0681 |
| 24.98 | 36.3308 |
| 22.8 | 26.3106 |
| 22.12 | 24.6248 |
| 21.12 | 22.0207 |
| 15.83 | 16.0276 |

**Figure S76.** Correlation between the experimental ^13^C NMR chemical shifts (x-axis) and the calculated values (y-axis) for compound **2**, RRSR isomer. The linear regression line is represented by the equation shown in the plot, along with the coefficient of determination (R^2^). The chemical shifts were calculated at the B3LYP/6-311+G(2d,p)//M06-2X/6-31+G(d,p) level of theory using Gaussian 16.

Carbon - SRSR

Calc

| Exp | SRSR |
| --- | --- |
| 167.13 | 176.026 |
| 157.11 | 166.897 |
| 135.66 | 144.48 |
| 132.1 | 145.945 |
| 129.69 | 137.343 |
| 129.69 | 137.604 |
| 124.93 | 132.162 |
| 124.93 | 132.23 |
| 98.8 | 102.811 |
| 81.54 | 97.7768 |
| 82.15 | 86.0212 |
| 48.04 | 53.5323 |
| 42.59 | 47.6991 |
| 33.89 | 38.8174 |
| 31.63 | 29.0434 |
| 24.98 | 37.7203 |
| 22.8 | 27.5358 |
| 22.12 | 23.8876 |
| 21.12 | 22.6665 |
| 15.83 | 17.8756 |

**Figure S77.** Correlation between the experimental ^13^C NMR chemical shifts (x-axis) and the calculated values (y-axis) for compound **2**, SRSR isomer. The linear regression line is represented by the equation shown in the plot, along with the coefficient of determination (R^2^). The chemical shifts were calculated at the B3LYP/6-311+G(2d,p)//M06-2X/6-31+G(d,p) level of theory using Gaussian 16.

Hydrogen - RRSR

Calc

| Exp | RRSR |
| --- | --- |
| 7.37 | 7.82381 |
| 7.37 | 7.81537 |
| 7.13 | 7.61813 |
| 7.13 | 7.59071 |
| 6.06 | 6.18843 |
| 3.43 | 3.54318 |
| 1.36 | 2.02918 |
| 2.23 | 1.39158 |
| 1.36 | 1.13226 |
| 1.58 | 1.56891 |
| 1.05 | 0.785023 |
| 0.8 | 0.591718 |
| 1.17 | 0.980245 |
| 0.88 | 0.930265 |
| 1.62 | 1.53924 |
| 0.91 | 0.846928 |
| 0.75 | 0.831926 |
| 0.5 | 0.82504 |

**Figure S78.** Correlation between the experimental ^1^H NMR chemical shifts (x-axis) and the calculated values (y-axis) for compound **2**, RRSR isomer. The linear regression line is represented by the equation shown in the plot, along with the coefficient of determination (R^2^). The chemical shifts were calculated at the B3LYP/6-311+G(2d,p)//M06-2X/6-31+G(d,p) level of theory using Gaussian 16.

Hydrogen - SRSR

Calc

| Exp | SRSR |
| --- | --- |
| 7.37 | 7.78857 |
| 7.37 | 7.78824 |
| 7.13 | 7.5053 |
| 7.13 | 7.51421 |
| 6.06 | 6.37803 |
| 3.43 | 3.44691 |
| 1.36 | 0.76932 |
| 2.23 | 2.31171 |
| 1.36 | 1.35719 |
| 1.58 | 1.54885 |
| 1.05 | 0.934464 |
| 0.8 | 0.743774 |
| 1.17 | 0.977533 |
| 0.88 | 0.884688 |
| 1.62 | 1.58978 |
| 0.91 | 0.903232 |
| 0.75 | 0.675713 |
| 0.5 | 0.434794 |

**Figure S79.** Correlation between the experimental ^1^H NMR chemical shifts (x-axis) and the calculated values (y-axis) for compound **2**, SRSR isomer. The linear regression line is represented by the equation shown in the plot, along with the coefficient of determination (R^2^). The chemical shifts were calculated at the B3LYP/6-311+G(2d,p)//M06-2X/6-31+G(d,p) level of theory using Gaussian 16.

Carbon - RRSR

Calc

| Exp | RRSR |
| --- | --- |
| 167.43 | 176.648 |
| 157.89 | 168.147 |
| 136.54 | 142.625 |
| 134.48 | 147.688 |
| 130.14 | 137.293 |
| 130.14 | 136.596 |
| 123.79 | 134.171 |
| 123.79 | 134.648 |
| 98.77 | 99.8238 |
| 80.11 | 94.0259 |
| 81.92 | 83.6937 |
| 48.02 | 52.7249 |
| 42.62 | 40.9781 |
| 33.93 | 38.7784 |
| 24.75 | 32.2314 |
| 31.63 | 37.0797 |
| 22.79 | 28.1441 |
| 21.02 | 23.4118 |
| 22.13 | 24.8131 |
| 21.19 | 21.2951 |
| 15.82 | 18.0373 |

**Figure S80.** Correlation between the experimental ^13^C NMR chemical shifts (x-axis) and the calculated values (y-axis) for compound **3**, RRSR isomer. The linear regression line is represented by the equation shown in the plot, along with the coefficient of determination (R^2^). The chemical shifts were calculated at the B3LYP/6-311+G(2d,p)//M06-2X/6-31+G(d,p) level of theory using Gaussian 16.

Carbon - SRSR

Calc

| Exp | SRSR |
| --- | --- |
| 167.43 | 176.272 |
| 157.89 | 167.76 |
| 136.54 | 142.904 |
| 134.48 | 146.463 |
| 130.14 | 137.801 |
| 130.14 | 137.674 |
| 123.79 | 131.383 |
| 123.79 | 131.565 |
| 98.77 | 102.863 |
| 80.11 | 95.9053 |
| 81.92 | 85.757 |
| 48.02 | 53.3516 |
| 42.62 | 47.7758 |
| 33.93 | 38.9213 |
| 24.75 | 28.46 |
| 31.63 | 37.9418 |
| 22.79 | 27.5559 |
| 21.02 | 23.4238 |
| 22.13 | 23.9025 |
| 21.19 | 23.1392 |
| 15.82 | 17.6732 |

**Figure S81.** Correlation between the experimental ^13^C NMR chemical shifts (x-axis) and the calculated values (y-axis) for compound **3**, SRSR isomer. The linear regression line is represented by the equation shown in the plot, along with the coefficient of determination (R^2^). The chemical shifts were calculated at the B3LYP/6-311+G(2d,p)//M06-2X/6-31+G(d,p) level of theory using Gaussian 16.

Hydrogen - RRSR

Calc

|  | RRSR |
| --- | --- |
| 7.19 | 7.6436 |
| 7.19 | 7.68148 |
| 7.07 | 7.53818 |
| 7.07 | 7.56262 |
| 6.07 | 6.1122 |
| 3.4 | 3.44226 |
| 2.37 | 2.48343 |
| 1.32 | 1.96153 |
| 2.22 | 0.931264 |
| 1.34 | 1.05155 |
| 1.55 | 1.50966 |
| 1.04 | 0.560569 |
| 1.6 | 1.50156 |
| 1.14 | 0.947473 |
| 0.84 | 0.956156 |
| 0.78 | 0.574745 |
| 0.9 | 0.688712 |
| 0.69 | 0.837385 |
| 0.43 | 0.866858 |

**Figure S82.** Correlation between the experimental ^1^H NMR chemical shifts (x-axis) and the calculated values (y-axis) for compound **3**, RRSR isomer. The linear regression line is represented by the equation shown in the plot, along with the coefficient of determination (R^2^). The chemical shifts were calculated at the B3LYP/6-311+G(2d,p)//M06-2X/6-31+G(d,p) level of theory using Gaussian 16.

Hydrogen - SRSR

| Exp | SRSR |
| --- | --- |
| 7.19 | 7.64578 |
| 7.19 | 7.65041 |
| 7.07 | 7.49319 |
| 7.07 | 7.48321 |
| 6.07 | 6.35388 |
| 3.4 | 3.4199 |
| 2.37 | 2.49075 |
| 1.32 | 0.645905 |
| 2.22 | 2.31369 |
| 1.34 | 1.35506 |
| 1.55 | 1.52533 |
| 1.04 | 0.883875 |
| 1.6 | 1.58373 |
| 1.14 | 0.893506 |
| 0.84 | 0.865669 |
| 0.78 | 0.727914 |
| 0.9 | 0.886089 |
| 0.69 | 0.398182 |
| 0.43 | 0.512839 |

**Figure S83.** Correlation between the experimental ^1^H NMR chemical shifts (x-axis) and the calculated values (y-axis) for compound **3**, SRSR isomer. The linear regression line is represented by the equation shown in the plot, along with the coefficient of determination (R^2^). The chemical shifts were calculated at the B3LYP/6-311+G(2d,p)//M06-2X/6-31+G(d,p) level of theory using Gaussian 16.

Carbon - RRSR

Calc

| Exp | RRSR |
| --- | --- |
| 167.34 | 176.772 |
| 157.48 | 167.765 |
| 137.11 | 145.606 |
| 129.61 | 134.286 |
| 129.61 | 136.227 |
| 126.43 | 136.089 |
| 123.45 | 133.503 |
| 123.45 | 134.302 |
| 98.83 | 99.3141 |
| 80.99 | 94.8663 |
| 82.03 | 83.0942 |
| 48.06 | 52.7029 |
| 42.55 | 41.5456 |
| 33.93 | 38.8034 |
| 31.63 | 31.9174 |
| 24.86 | 37.1755 |
| 22.81 | 27.7507 |
| 22.13 | 24.0503 |
| 21.2 | 21.4427 |
| 15.83 | 17.6715 |

**Figure S84.** Correlation between the experimental ^13^C NMR chemical shifts (x-axis) and the calculated values (y-axis) for compound **4**, RRSR isomer. The linear regression line is represented by the equation shown in the plot, along with the coefficient of determination (R^2^). The chemical shifts were calculated at the B3LYP/6-311+G(2d,p)//M06-2X/6-31+G(d,p) level of theory using Gaussian 16.

Carbon - SRSR

Calc

| Exp | SRSR |
| --- | --- |
| 167.34 | 176.546 |
| 157.48 | 166.608 |
| 137.11 | 145.101 |
| 129.61 | 134.158 |
| 129.61 | 136.167 |
| 126.43 | 136.277 |
| 123.45 | 133.689 |
| 123.45 | 132.604 |
| 98.83 | 103.128 |
| 80.99 | 96.186 |
| 82.03 | 85.1576 |
| 48.06 | 53.9387 |
| 42.55 | 47.6476 |
| 33.93 | 38.8636 |
| 31.63 | 29.6637 |
| 24.86 | 37.7183 |
| 22.81 | 27.1548 |
| 22.13 | 23.8583 |
| 21.2 | 23.038 |
| 15.83 | 17.3664 |

**Figure S85.** Correlation between the experimental ^13^C NMR chemical shifts (x-axis) and the calculated values (y-axis) for compound **4**, SRSR isomer. The linear regression line is represented by the equation shown in the plot, along with the coefficient of determination (R^2^). The chemical shifts were calculated at the B3LYP/6-311+G(2d,p)//M06-2X/6-31+G(d,p) level of theory using Gaussian 16.

Hydrogen - RRSR

Calc

| Exp | RRSR |
| --- | --- |
| 7.4 | 7.8283 |
| 7.4 | 7.82544 |
| 7.28 | 7.74959 |
| 7.17 | 7.67282 |
| 7.17 | 7.65738 |
| 6.12 | 6.18752 |
| 3.41 | 3.53947 |
| 1.37 | 2.00425 |
| 2.23 | 1.23577 |
| 1.39 | 1.12978 |
| 1.56 | 1.55338 |
| 1.06 | 0.721951 |
| 1.39 | 0.603944 |
| 1.17 | 1.00162 |
| 0.8 | 0.989953 |
| 1.58 | 1.53266 |
| 0.91 | 0.716451 |
| 0.71 | 0.870159 |
| 0.43 | 0.856283 |

**Figure S86.** Correlation between the experimental ^1^H NMR chemical shifts (x-axis) and the calculated values (y-axis) for compound **4**, RRSR isomer. The linear regression line is represented by the equation shown in the plot, along with the coefficient of determination (R^2^). The chemical shifts were calculated at the B3LYP/6-311+G(2d,p)//M06-2X/6-31+G(d,p) level of theory using Gaussian 16.

Hydrogen - SRSR

Calc

| Exp | SRSR |
| --- | --- |
| 7.4 | 7.80807 |
| 7.4 | 7.81773 |
| 7.28 | 7.75344 |
| 7.17 | 7.62701 |
| 7.17 | 7.65186 |
| 6.12 | 6.26468 |
| 3.41 | 3.60228 |
| 1.37 | 1.34241 |
| 2.23 | 2.27287 |
| 1.39 | 1.42408 |
| 1.56 | 1.63272 |
| 1.06 | 0.965668 |
| 1.39 | 0.777064 |
| 1.17 | 1.09276 |
| 0.8 | 0.954537 |
| 1.58 | 1.63054 |
| 0.91 | 0.910691 |
| 0.71 | 0.705363 |
| 0.43 | 0.6217 |

**Figure S87.** Correlation between the experimental ^1^H NMR chemical shifts (x-axis) and the calculated values (y-axis) for compound **4**, SRSR isomer. The linear regression line is represented by the equation shown in the plot, along with the coefficient of determination (R^2^). The chemical shifts were calculated at the B3LYP/6-311+G(2d,p)//M06-2X/6-31+G(d,p) level of theory using Gaussian 16.

Carbon - RRSR

Calc

| Exp | RRSR |
| --- | --- |
| 167.3 | 176.441 |
| 162.39 | 172.495 |
| 157.57 | 168.135 |
| 132.95 | 141.427 |
| 126.22 | 137.396 |
| 126.31 | 136.542 |
| 116.25 | 122.92 |
| 116.48 | 122.735 |
| 98.75 | 98.9478 |
| 80.37 | 94.5554 |
| 82.01 | 83.0163 |
| 48.05 | 52.5408 |
| 42.59 | 40.8379 |
| 33.9 | 38.7584 |
| 24.97 | 30.9992 |
| 31.64 | 37.1746 |
| 22.8 | 26.6953 |
| 22.11 | 24.1186 |
| 21.15 | 21.9662 |
| 15.85 | 16.3588 |

**Figure S88.** Correlation between the experimental ^13^C NMR chemical shifts (x-axis) and the calculated values (y-axis) for compound **5**, RRSR isomer. The linear regression line is represented by the equation shown in the plot, along with the coefficient of determination (R^2^). The chemical shifts were calculated at the B3LYP/6-311+G(2d,p)//M06-2X/6-31+G(d,p) level of theory using Gaussian 16.

Carbon - SRSR

Calc

| Exp | SRSR |
| --- | --- |
| 167.3 | 176.179 |
| 162.39 | 171.756 |
| 157.57 | 167.777 |
| 132.95 | 141.761 |
| 126.22 | 134.771 |
| 126.31 | 134.623 |
| 116.25 | 123.508 |
| 116.48 | 123.383 |
| 98.75 | 102.356 |
| 80.37 | 96.2118 |
| 82.01 | 85.0726 |
| 48.05 | 53.3659 |
| 42.59 | 48.0066 |
| 33.9 | 38.6963 |
| 24.97 | 28.594 |
| 31.64 | 37.5336 |
| 22.8 | 27.1183 |
| 22.11 | 23.786 |
| 21.15 | 23.006 |
| 15.85 | 17.1582 |

**Figure S89.** Correlation between the experimental ^13^C NMR chemical shifts (x-axis) and the calculated values (y-axis) for compound **5**, SRSR isomer. The linear regression line is represented by the equation shown in the plot, along with the coefficient of determination (R^2^). The chemical shifts were calculated at the B3LYP/6-311+G(2d,p)//M06-2X/6-31+G(d,p) level of theory using Gaussian 16.

Hydrogen - RRSR

Calc

| Exp | RRSR |
| --- | --- |
| 7.19 | 7.66226 |
| 7.19 | 7.63765 |
| 7.1 | 7.52919 |
| 7.1 | 7.52983 |
| 6.01 | 6.13487 |
| 3.43 | 3.53836 |
| 1.37 | 2.03035 |
| 2.21 | 1.30124 |
| 1.37 | 1.12239 |
| 1.58 | 1.54246 |
| 1.05 | 0.668007 |
| 0.83 | 0.600787 |
| 1.18 | 0.973415 |
| 0.83 | 0.956878 |
| 1.62 | 1.54347 |
| 0.91 | 0.748289 |
| 0.75 | 0.848485 |
| 0.52 | 0.82964 |

**Figure S90.** Correlation between the experimental ^1^H NMR chemical shifts (x-axis) and the calculated values (y-axis) for compound **5**, RRSR isomer. The linear regression line is represented by the equation shown in the plot, along with the coefficient of determination (R^2^). The chemical shifts were calculated at the B3LYP/6-311+G(2d,p)//M06-2X/6-31+G(d,p) level of theory using Gaussian 16.

Hydrogen - SRSR

Calc

| Exp | SRSR |
| --- | --- |
| 7.19 | 7.58741 |
| 7.19 | 7.59073 |
| 7.1 | 7.51546 |
| 7.1 | 7.51048 |
| 6.01 | 6.25108 |
| 3.43 | 3.47335 |
| 1.37 | 0.646398 |
| 2.21 | 2.21091 |
| 1.37 | 1.38543 |
| 1.58 | 1.564 |
| 1.05 | 0.891265 |
| 0.83 | 0.730863 |
| 1.18 | 0.914978 |
| 0.83 | 0.877808 |
| 1.62 | 1.5665 |
| 0.91 | 0.886001 |
| 0.75 | 0.597185 |
| 0.52 | 0.476901 |

**Figure S91.** Correlation between the experimental ^1^H NMR chemical shifts (x-axis) and the calculated values (y-axis) for compound **5**, SRSR isomer. The linear regression line is represented by the equation shown in the plot, along with the coefficient of determination (R^2^). The chemical shifts were calculated at the B3LYP/6-311+G(2d,p)//M06-2X/6-31+G(d,p) level of theory using Gaussian 16.

Carbon - RRSR

Calc

| Exp | RRSR |
| --- | --- |
| 167.46 | 176.732 |
| 158.47 | 168.154 |
| 158.23 | 168.38 |
| 129.66 | 136.447 |
| 126.2 | 137.384 |
| 126.2 | 137.353 |
| 114.57 | 123.707 |
| 114.57 | 114.839 |
| 98.67 | 98.8387 |
| 79.16 | 93.1119 |
| 81.77 | 82.3484 |
| 55.47 | 56.8097 |
| 48.03 | 52.7969 |
| 42.63 | 41.8096 |
| 33.91 | 38.7192 |
| 24.75 | 31.3526 |
| 31.63 | 36.8622 |
| 22.76 | 27.0074 |
| 22.08 | 24.3294 |
| 21.2 | 21.8045 |
| 15.88 | 16.8618 |

**Figure S92.** Correlation between the experimental ^13^C NMR chemical shifts (x-axis) and the calculated values (y-axis) for compound **6**, RRSR isomer. The linear regression line is represented by the equation shown in the plot, along with the coefficient of determination (R^2^). The chemical shifts were calculated at the B3LYP/6-311+G(2d,p)//M06-2X/6-31+G(d,p) level of theory using Gaussian 16.

Carbon - SRSR

Calc

| Exp | SRSR |
| --- | --- |
| 167.46 | 176.391 |
| 158.47 | 168.31 |
| 158.23 | 167.547 |
| 129.66 | 137.165 |
| 126.2 | 135.197 |
| 126.2 | 134.999 |
| 114.57 | 124.658 |
| 114.57 | 115.593 |
| 98.67 | 102.281 |
| 79.16 | 94.2876 |
| 81.77 | 84.5047 |
| 55.47 | 56.6066 |
| 48.03 | 53.325 |
| 42.63 | 47.7624 |
| 33.91 | 38.916 |
| 24.75 | 28.4358 |
| 31.63 | 37.8708 |
| 22.76 | 27.1291 |
| 22.08 | 23.817 |
| 21.2 | 22.9242 |
| 15.88 | 17.2881 |

**Figure S93.** Correlation between the experimental ^13^C NMR chemical shifts (x-axis) and the calculated values (y-axis) for compound **6**, SRSR isomer. The linear regression line is represented by the equation shown in the plot, along with the coefficient of determination (R^2^). The chemical shifts were calculated at the B3LYP/6-311+G(2d,p)//M06-2X/6-31+G(d,p) level of theory using Gaussian 16.

Hydrogen - RRSR

Calc

| Exp | RRSR |
| --- | --- |
| 7.11 | 7.57555 |
| 7.11 | 7.64514 |
| 6.89 | 7.32991 |
| 6.89 | 7.20411 |
| 5.97 | 6.10442 |
| 3.81 | 3.91947 |
| 3.39 | 3.63553 |
| 1.29 | 2.07449 |
| 2.19 | 1.61325 |
| 1.33 | 1.21831 |
| 1.57 | 1.61535 |
| 1.01 | 0.887574 |
| 0.79 | 0.686823 |
| 1.12 | 1.07414 |
| 0.82 | 1.01181 |
| 1.57 | 1.56977 |
| 0.88 | 0.81587 |
| 0.69 | 0.868161 |
| 0.47 | 0.860977 |

**Figure S94.** Correlation between the experimental ^1^H NMR chemical shifts (x-axis) and the calculated values (y-axis) for compound **6**, RRSR isomer. The linear regression line is represented by the equation shown in the plot, along with the coefficient of determination (R^2^). The chemical shifts were calculated at the B3LYP/6-311+G(2d,p)//M06-2X/6-31+G(d,p) level of theory using Gaussian 16.

Hydrogen - SRSR

Calc

| Exp | SRSR |
| --- | --- |
| 7.11 | 7.51323 |
| 7.11 | 7.57809 |
| 6.89 | 7.33006 |
| 6.89 | 7.21103 |
| 5.97 | 6.19504 |
| 3.81 | 3.94149 |
| 3.39 | 3.51121 |
| 1.29 | 0.645408 |
| 2.19 | 2.15948 |
| 1.33 | 1.38638 |
| 1.57 | 1.547 |
| 1.01 | 0.877876 |
| 0.79 | 0.722124 |
| 1.12 | 0.904862 |
| 0.82 | 0.870837 |
| 1.57 | 1.58157 |
| 0.88 | 0.879749 |
| 0.69 | 0.565166 |
| 0.47 | 0.514594 |

**Figure S95.** Correlation between the experimental ^1^H NMR chemical shifts (x-axis) and the calculated values (y-axis) for compound **6**, SRSR isomer. The linear regression line is represented by the equation shown in the plot, along with the coefficient of determination (R^2^). The chemical shifts were calculated at the B3LYP/6-311+G(2d,p)//M06-2X/6-31+G(d,p) level of theory using Gaussian 16.

Carbon - RRSR

Calc

| Exp | RRSR |
| --- | --- |
| 167.25 | 179.216 |
| 160.61 | 169.963 |
| 157.43 | 170.359 |
| 138.32 | 147.888 |
| 130.38 | 137.492 |
| 115.38 | 124.59 |
| 111.9 | 121.611 |
| 109.24 | 117.084 |
| 98.89 | 100.881 |
| 81.4 | 93.8736 |
| 82.13 | 84.4775 |
| 55.41 | 57.6688 |
| 48.06 | 53.2521 |
| 42.59 | 42.8879 |
| 33.93 | 39.1141 |
| 24.84 | 31.9148 |
| 31.63 | 37.6398 |
| 22.79 | 27.4239 |
| 22.13 | 24.4504 |
| 21.2 | 22.2991 |
| 15.74 | 17.1943 |

**Figure S96.** Correlation between the experimental ^13^C NMR chemical shifts (x-axis) and the calculated values (y-axis) for compound **7**, RRSR isomer. The linear regression line is represented by the equation shown in the plot, along with the coefficient of determination (R^2^). The chemical shifts were calculated at the B3LYP/6-311+G(2d,p)//M06-2X/6-31+G(d,p) level of theory using Gaussian 16.

Carbon - SRSR

Calc

| Exp | SRSR |
| --- | --- |
| 167.25 | 179.03 |
| 160.61 | 169.569 |
| 157.43 | 168.875 |
| 138.32 | 147.176 |
| 130.38 | 137.814 |
| 115.38 | 124.336 |
| 111.9 | 117.881 |
| 109.24 | 118.53 |
| 98.89 | 104.402 |
| 81.4 | 95.2777 |
| 82.13 | 86.4422 |
| 55.41 | 57.6227 |
| 48.06 | 54.421 |
| 42.59 | 48.7981 |
| 33.93 | 39.393 |
| 24.84 | 30.2655 |
| 31.63 | 38.3592 |
| 22.79 | 27.6597 |
| 22.13 | 24.3079 |
| 21.2 | 23.2765 |
| 15.74 | 17.8316 |

**Figure S97.** Correlation between the experimental ^13^C NMR chemical shifts (x-axis) and the calculated values (y-axis) for compound **7**, SRSR isomer. The linear regression line is represented by the equation shown in the plot, along with the coefficient of determination (R^2^). The chemical shifts were calculated at the B3LYP/6-311+G(2d,p)//M06-2X/6-31+G(d,p) level of theory using Gaussian 16.

Hydrogen - RRSR

Calc

| Exp | RRSR |
| --- | --- |
| 7.3 | 7.71483 |
| 6.81 | 7.26972 |
| 6.74 | 7.21361 |
| 6.7 | 7.24743 |
| 6.13 | 6.47796 |
| 3.84 | 3.9743 |
| 3.42 | 3.54639 |
| 1.38 | 1.97059 |
| 2.25 | 1.18839 |
| 1.41 | 1.12517 |
| 1.59 | 1.53678 |
| 1.05 | 0.704748 |
| 0.79 | 0.569975 |
| 1.17 | 0.970557 |
| 0.86 | 0.937187 |
| 1.59 | 1.48306 |
| 0.91 | 0.718113 |
| 0.71 | 0.830983 |
| 0.44 | 0.79537 |

**Figure S98.** Correlation between the experimental ^1^H NMR chemical shifts (x-axis) and the calculated values (y-axis) for compound **7**, RRSR isomer. The linear regression line is represented by the equation shown in the plot, along with the coefficient of determination (R^2^). The chemical shifts were calculated at the B3LYP/6-311+G(2d,p)//M06-2X/6-31+G(d,p) level of theory using Gaussian 16.

Hydrogen - SRSR

Calc

| Exp | SRSR |
| --- | --- |
| 7.3 | 7.72828 |
| 6.81 | 7.29193 |
| 6.74 | 7.21858 |
| 6.7 | 7.1932 |
| 6.13 | 6.49597 |
| 3.84 | 3.9566 |
| 3.42 | 3.65587 |
| 1.38 | 1.16931 |
| 2.25 | 2.18876 |
| 1.41 | 1.44328 |
| 1.59 | 1.58801 |
| 1.05 | 0.939225 |
| 0.79 | 0.776703 |
| 1.17 | 1.06388 |
| 0.86 | 0.985297 |
| 1.59 | 1.59145 |
| 0.91 | 0.883508 |
| 0.71 | 0.680541 |
| 0.44 | 0.568482 |

**Figure S99.** Correlation between the experimental ^1^H NMR chemical shifts (x-axis) and the calculated values (y-axis) for compound **7**, SRSR isomer. The linear regression line is represented by the equation shown in the plot, along with the coefficient of determination (R^2^). The chemical shifts were calculated at the B3LYP/6-311+G(2d,p)//M06-2X/6-31+G(d,p) level of theory using Gaussian 16.

**Figure S100.** DP4 results for compound **1**.

**Figure S101.** DP4+ results for compound **1**.

**Figure S102.** DP4 results for compound **2**.

**Figure S103.** DP4+ results for compound **2**.

**Figure S104.** DP4 results for compound **3**.

**Figure S105.** DP4+ results for compound **3**.

**Figure S106.** DP4 results for compound **4**.

**Figure S107.** DP4+ results for compound **4**.

**Figure S108.** DP4 results for compound **5**.

**Figure S109.** DP4+ results for compound **5**.

**Figure S110.** DP4 results for compound **6**.

**Figure S111.** DP4+ results for compound **6**.

**Figure S112.** DP4 results for compound **7**.

**Figure S113.** DP4+ results for compound **7**.

Cucumis sativus

Root inhibition (%)

| Compunds | 50 µM | 75 µM | 150 µM | 300 µM | 500 µM |
| --- | --- | --- | --- | --- | --- |
| 1b | -26.6494 | -46.1675 | -46.5668 | -44.6078 | -20.7355 |
| 2b | 4.55045 | -8.27038 | -2.93624 | -29.8322 | -16.5175 |
| 3b | -27.182 | -32.6299 | -27.0422 | 0.42184 | -22.7325 |
| 4b | -18.0234 | -28.1731 | -30.8612 | -24.1741 | -17.1391 |
| 5b | -7.82261 | -18.0811 | -16.0051 | -39.8739 | -32.2436 |
| 6b | -34.3517 | -43.0826 | -2.6038 | 1.65291 | -7.73979 |
| 7b | 10.1555 | -50.793 | -16.9185 | 9.04517 | -34.347 |
| DUAL | -70.0018 | -79.1143 | -72.3347 | -84.806 | -90.6077 |

**Figure S114.** Development of the roots of *C. sativus*.

Cucumis sativus

Shoot inhibition (%)

| Compounds | 50 µM | 75 µM | 150 µM | 300 µM | 500 µM |
| --- | --- | --- | --- | --- | --- |
| 1b | -39.5376 | -45.6569 | -61.3866 | -67.3734 | -36.3252 |
| 2b | -0.698915 | -24.7948 | -14.1869 | -40.6077 | -32.1944 |
| 3b | -35.1414 | -41.6745 | -44.5019 | -23.4295 | -45.8964 |
| 4b | -10.7793 | -24.9394 | -38.7354 | -25.8089 | -27.0069 |
| 5b | -25.4498 | -16.6649 | -21.6492 | -45.1761 | -51.491 |
| 6b | -51.2879 | -53.197 | -24.1247 | -10.8486 | -7.6216 |
| 7b | -11.5141 | -59.8855 | -26.2486 | -9.99256 | -47.3954 |
| DUAL | -67.6907 | -76.3029 | -71.0055 | -79.2554 | -87.7874 |

**Figure S115.** Development of the shoots of *C. sativus*.

**Table S1.** Development of root parts of cucumber plants in relation to the control.

| **Inhibition: Root** | | | | | |
| --- | --- | --- | --- | --- | --- |
| **Substances** | **50 µM** | **75 µM** | **150 µM** | **300 µM** | **500 µM** |
| **DUAL** | -70.00 | -79.11 | -72.33 | -84.81 | -90.61 |
| **1b** | -26.65 | -46.17 | -46.57 | -44.61 | -20.74 |
| **2b** | 4.55 | -8.27 | -2.94 | -29.83 | -16.52 |
| **3b** | -27.18 | -32.63 | -27.04 | 0.42 | -22.73 |
| **4b** | -18.02 | -28.17 | -30.86 | -24.17 | -17.14 |
| **5b** | -7.82 | -18.08 | -16.01 | -39.87 | -32.24 |
| **6b** | -34.35 | -43.08 | -2.60 | 1.65 | -7.74 |
| **7b** | 10.16 | -50.79 | -16.92 | 9.05 | -34.35 |

**Table S2.** Development of shoot of cucumber plants in relation to the control.

| **Inhibition: shoot** | | | | | |
| --- | --- | --- | --- | --- | --- |
| **Substances** | **50 µM** | **75 µM** | **150 µM** | **300 µM** | **500 µM** |
| **DUAL** | -67.69 | -76.30 | -71.01 | -79.26 | -87.79 |
| **1b** | -39.54 | -45.66 | -61.39 | -67.37 | -36.33 |
| **2b** | -0.70 | -24.79 | -14.19 | -40.61 | -32.19 |
| **3b** | -35.14 | -41.67 | -44.50 | -23.43 | -45.90 |
| **4b** | -10.78 | -24.94 | -38.74 | -25.81 | -27.01 |
| **5b** | -25.45 | -16.66 | -21.65 | -45.18 | -51.49 |
| **6b** | -51.29 | -53.20 | -24.12 | -10.85 | -7.62 |
| **7b** | -11.51 | -59.89 | -26.25 | -9.99 | -47.40 |

Solanum lycopersicum

Root inhibition (%)

| Compounds | 50 µM | 75 µM | 150 µM | 250 µM | 500 µM |
| --- | --- | --- | --- | --- | --- |
| 1b | -8.49069 | 26.0071 | 25.9204 | 7.61284 | 23.528 |
| 2b | 4.97852 | 20.4842 | 0.426494 | 12.9704 | 43.2733 |
| 3b | 45.4668 | 32.8822 | 0.301409 | 25.7034 | 29.89 |
| 4b | 36.3319 | 41.5583 | 26.0101 | 32.6878 | 42.3713 |
| 5b | 26.3002 | 10.1289 | 39.7521 | 24.2747 | 36.6905 |
| 6b | 4.09992 | 3.70281 | 2.8988 | 4.71479 | 33.5679 |
| 7b | 2.32085 | 2.28242 | -10.6721 | -14.9348 | -19.4416 |
| DUAL | -38.9526 | -43.1053 | -47.646 | -55.9039 | -59.4989 |

**Figure S116.** Development of the roots of *S. lycopersicum*.

Solanum lycopersicum

Shoot inhibition (%)

| Compounds | 50 µM | 75 µM | 150 µM | 300 µM | 500 µM |
| --- | --- | --- | --- | --- | --- |
| 1b | -0.726353 | 23.4375 | 17.3928 | 2.55534 | 2.98832 |
| 2b | 9.42983 | 14.6978 | 5.11848 | 2.13867 | 9.19173 |
| 3b | 18.4898 | 10.6551 | 6.54284 | 15.8983 | 7.38541 |
| 4b | 6.64205 | 15.308 | 7.87792 | 21.0536 | 18.1716 |
| 5b | 9.61408 | 2.07489 | 23.5856 | 4.67488 | 20.8764 |
| 6b | 12.0511 | 14.3109 | 3.88191 | 6.79937 | 16.2214 |
| 7b | 8.71056 | 12.0369 | -5.92846 | 7.60013 | -7.49313 |
| DUAL | -37.4826 | -29.7939 | -50.8667 | -51.5087 | -52.3931 |

**Figure S117.** Development of the shoots of *S. lycopersicum*.

**Table S3.** Development of the root of tomato plants in relation to the control.

| **Inhibition: root** | | | | | |
| --- | --- | --- | --- | --- | --- |
| **Substances** | **50 µM** | **75 µM** | **150 µM** | **300 µM** | **500 µM** |
| **DUAL** | -38.95 | -43.11 | -47.65 | -55.90 | -59.50 |
| **1b** | -8.49 | 26.01 | 25.92 | 7.61 | 23.53 |
| **2b** | 4.98 | 20.48 | 0.43 | 12.97 | 43.27 |
| **3b** | 45.47 | 32.88 | 0.30 | 25.70 | 29.89 |
| **4b** | 36.33 | 41.56 | 26.01 | 32.69 | 42.37 |
| **5b** | 26.30 | 10.13 | 39.75 | 24.27 | 36.69 |
| **6b** | 4.10 | 3.70 | 2.90 | 4.71 | 33.57 |
| **7b** | 2.32 | 2.28 | -10.67 | -14.93 | -19.44 |

**Table S4.** Development of the shoot of tomato plants in relation to the control.

| **Inhibition: shoot** | | | | | |
| --- | --- | --- | --- | --- | --- |
| **Substances** | **50 µM** | **75 µM** | **150 µM** | **300 µM** | **500 µM** |
| **DUAL** | -37.48 | -29.79 | -50.87 | -51.51 | -52.39 |
| **1b** | -0.73 | 23.44 | 17.39 | 2.56 | 2.99 |
| **2b** | 9.43 | 14.70 | 5.12 | 2.14 | 9.19 |
| **3b** | 18.49 | 10.66 | 6.54 | 15.90 | 7.39 |
| **4b** | 6.64 | 15.31 | 7.88 | 21.05 | 18.17 |
| **5b** | 9.61 | 2.07 | 23.59 | 4.67 | 20.88 |
| **6b** | 12.05 | 14.31 | 3.88 | 6.80 | 16.22 |
| **7b** | 8.71 | 12.04 | -5.93 | 7.60 | -7.49 |

Sorghum bicolor

Root inhibition (%)

| Compounds | 50 µM | 125 µM | 250 µM | 500 µM | 1000 µM |
| --- | --- | --- | --- | --- | --- |
| 1b | 40.326 | 46.8191 | 19.0229 | 13.9067 | -12.3501 |
| 2b | 26.1765 | 10.6537 | 9.52401 | 27.167 | 68.0775 |
| 3b | -10.6145 | -17.8601 | -13.0303 | 10.9812 | 24.2182 |
| 4b | 25.4693 | 11.7564 | 32.2899 | 17.1486 | 20.0379 |
| 5b | 7.3799 | 17.3743 | -1.86629 | -16.3458 | 31.3221 |
| 6b | 34.8124 | 29.5135 | 50.5713 | 34.58 | 8.73898 |
| 7b | -26.959 | -2.37472 | 44.8694 | -4.49123 | 26.4991 |
| DUAL | -56.1806 | -68.619 | -78.159 | -88.9256 | -98.7372 |

**Figure S118.** Development of the roots of *S. bicolor*.

**Figure S119.** Development of the shoots of *S. bicolor*.

**Table S5.** Development of the root of sorghum plants in relation to the control.

| **Inhibition: root** | | | | | |
| --- | --- | --- | --- | --- | --- |
| **Substances** | **50 µM** | **75 µM** | **150 µM** | **300 µM** | **500 µM** |
| **DUAL** | -56.18 | -68.62 | -78.16 | -88.93 | -98.74 |
| **1b** | 40.33 | 46.82 | 19.02 | 13.91 | -12.35 |
| **2b** | 26.18 | 10.65 | 9.52 | 27.17 | 68.08 |
| **3b** | -10.61 | -17.86 | -13.03 | 10.98 | 24.22 |
| **4b** | 25.47 | 11.76 | 32.29 | 17.15 | 20.04 |
| **5b** | 7.38 | 17.37 | -1.87 | -16.35 | 31.32 |
| **6b** | 34.81 | 29.51 | 50.57 | 34.58 | 8.74 |
| **7b** | -26.96 | -2.37 | 44.87 | -4.49 | 26.50 |

**Table S6.** Development of the shoot of sorghum plants in relation to the control.

| **Inhibition: shoot** | | | | | |
| --- | --- | --- | --- | --- | --- |
| **Substances** | **50 µM** | **75 µM** | **150 µM** | **300 µM** | **500 µM** |
| **DUAL** | -60.56 | -69.64 | -69.57 | -79.44 | -92.88 |
| **1b** | -11.90 | 9.88 | 12.33 | -11.66 | -13.76 |
| **2b** | -1.69 | -6.75 | -18.05 | -6.22 | 27.89 |
| **3b** | -2.55 | -14.79 | -20.88 | -7.07 | -1.38 |
| **4b** | 5.48 | 3.39 | -2.17 | 5.45 | 0.12 |
| **5b** | -11.98 | -0.01 | -30.41 | -37.34 | -3.59 |
| **6b** | -7.33 | -11.13 | 6.52 | -10.57 | -28.17 |
| **7b** | -20.78 | 6.31 | 22.68 | -15.81 | 1.54 |

**Table S7.** Development of the root of brachiaria plants in relation to the control.

| **Inhibition: root** | | | | | |
| --- | --- | --- | --- | --- | --- |
| **Substances** | **50 µM** | **75 µM** | **150 µM** | **300 µM** | **500 µM** |
| **DUAL** | -24.34 | -49.83 | -65.96 | -86.30 | -94.00 |
| **1b** | -44.64 | -55.96 | -45.80 | -65.85 | -56.41 |
| **2b** | -27.30 | -38.72 | -30.33 | -34.11 | -8.67 |
| **3b** | -26.80 | -45.76 | -15.48 | -59.71 | 10.46 |
| **4b** | -32.57 | -41.76 | -22.68 | -63.10 | -57.60 |
| **5b** | -31.77 | -29.35 | -58.66 | -64.52 | -64.43 |
| **6b** | -11.80 | -48.78 | -29.43 | -29.76 | -27.49 |
| **7b** | -32.25 | -49.46 | -29.72 | -59.07 | -42.12 |

**Table S8.** Development of the shoot of brachiaria plants in relation to the control.

| **Inhibition: shoot** | | | | | |
| --- | --- | --- | --- | --- | --- |
| **Substances** | **50 µM** | **75 µM** | **150 µM** | **300 µM** | **500 µM** |
| **DUAL** | -78.37 | -85.97 | -87.79 | -93.42 | -95.92 |
| **1b** | -27.35 | -36.52 | -33.42 | -69.23 | -48.44 |
| **2b** | -23.73 | -37.54 | -34.15 | -27.61 | -20.04 |
| **3b** | -17.46 | -38.73 | -18.96 | -47.76 | -5.54 |
| **4b** | -24.07 | -38.19 | -24.35 | -49.88 | -40.49 |
| **5b** | -29.92 | -25.29 | -47.68 | -65.02 | -52.31 |
| **6b** | -3.41 | -52.22 | -19.47 | -32.70 | -22.56 |
| **7b** | -31.33 | -49.49 | -26.74 | -54.40 | -34.64 |

Bidens pilosa

Root inhibition (%)

| Compounds | 50 µM | 75 µM | 150 µM | 300 µM | 500 µM |
| --- | --- | --- | --- | --- | --- |
| 1b | -7.72099 | -15.6635 | -7.25044 | -21.2814 | -7.93871 |
| 2b | -29.2316 | -27.3928 | -17.0911 | -23.3673 | -22.6707 |
| 3b | -24.8536 | -33.0643 | -51.8148 | -29.201 | -29.6766 |
| 4b | -33.2488 | -44.362 | -50.9133 | -23.8583 | -35.0685 |
| 5b | -47.7504 | -34.0956 | -49.3657 | -40.0958 | -61.5668 |
| 6b | -40.8472 | -42.9874 | -46.5385 | -36.0664 | -36.8983 |
| 7b | -44.0294 | -47.1508 | -51.1713 | -40.2656 | -58.7263 |
| DUAL | -56.6391 | -66.6286 | -60.3301 | -71.4235 | -86.1759 |

**Figure S120.** Development of the roots of B. pilosa.

Bidens pilosa

Shoot inhibition (%)

| Compounds | 50 µM | 75 µM | 150 µM | 300 µM | 500 µM |
| --- | --- | --- | --- | --- | --- |
| 1b | -0.183174 | -1.7504 | 7.99978 | -1.53456 | 3.59912 |
| 2b | -11.8714 | -6.31631 | -0.794822 | -12.7607 | -12.5718 |
| 3b | -11.0731 | -17.855 | -34.2782 | -10.4864 | -22.3437 |
| 4b | -20.1479 | -26.8914 | -38.0852 | -7.83326 | -26.5359 |
| 5b | -33.9855 | -24.2017 | -35.8874 | -22.01 | -38.5367 |
| 6b | -29.2044 | -22.77 | -27.7573 | -16.9852 | -14.3616 |
| 7b | -19.095 | -24.1214 | -21.5944 | -6.28653 | -35.8038 |
| DUAL | -47.479 | -66.5576 | -58.0906 | -71.5151 | -85.145 |

**Figure S121.** Development of the shoots of B. pilosa.

**Table S9.** Development of the root of beggartick plants in relation to the control.

| **Inhibition: root** | | | | | |
| --- | --- | --- | --- | --- | --- |
| **Substances** | **50 µM** | **75 µM** | **150 µM** | **300 µM** | **500 µM** |
| **DUAL** | -56.64 | -66.63 | -60.33 | -71.42 | -86.18 |
| **1b** | -7.72 | -15.66 | -7.25 | -21.28 | -7.94 |
| **2b** | -29.23 | -27.39 | -17.09 | -23.37 | -22.67 |
| **3b** | -24.85 | -33.06 | -51.81 | -29.20 | -29.68 |
| **4b** | -33.25 | -44.36 | -50.91 | -23.86 | -35.07 |
| **5b** | -47.75 | -34.10 | -49.37 | -40.10 | -61.57 |
| **6b** | -40.85 | -42.99 | -46.54 | -36.07 | -36.90 |
| **7b** | -44.03 | -47.15 | -51.17 | -40.27 | -58.73 |

**Table S10.** Development of the shoot of beggartick plants in relation to the control.

| **Inhibition: shoot** | | | | | |
| --- | --- | --- | --- | --- | --- |
| **Substances** | **50 µM** | **75 µM** | **150 µM** | **300 µM** | **500 µM** |
| **DUAL** | -47.48 | -66.56 | -58.09 | -71.52 | -85.14 |
| **1b** | -0.18 | -1.75 | 8.00 | -1.53 | 3.60 |
| **2b** | -11.87 | -6.32 | -0.79 | -12.76 | -12.57 |
| **3b** | -11.07 | -17.86 | -34.28 | -10.49 | -22.34 |
| **4b** | -20.15 | -26.89 | -38.09 | -7.83 | -26.54 |
| **5b** | -33.99 | -24.20 | -35.89 | -22.01 | -38.54 |
| **6b** | -29.20 | -22.77 | -27.76 | -16.99 | -14.36 |
| **7b** | -19.09 | -24.12 | -21.59 | -6.29 | -35.80 |
